# Diet and ADHD, Reviewing the Evidence: A Systematic Review of Meta-Analyses of Double-Blind Placebo-Controlled Trials Evaluating the Efficacy of Diet Interventions on the Behavior of Children with ADHD

**DOI:** 10.1371/journal.pone.0169277

**Published:** 2017-01-25

**Authors:** Lidy M. Pelsser, Klaas Frankena, Jan Toorman, Rob Rodrigues Pereira

**Affiliations:** 1 ADHD Research Centre, Eindhoven, the Netherlands; 2 Quantitative Veterinary Epidemiology group, Wageningen University & Research, Wageningen, the Netherlands; 3 Retired paediatrician, previously Catharina Hospital, Eindhoven, the Netherlands; 4 Medical Center Kinderplein, Rotterdam, the Netherlands; Chiba Daigaku, JAPAN

## Abstract

**Introduction:**

Attention-deficit/hyperactivity disorder (ADHD) is a debilitating mental health problem hampering the child’s development. The underlying causes include both genetic and environmental factors and may differ between individuals. The efficacy of diet treatments in ADHD was recently evaluated in three reviews, reporting divergent and confusing conclusions based on heterogeneous studies and subjects. To address this inconsistency we conducted a systematic review of meta-analyses of double-blind placebo-controlled trials evaluating the effect of diet interventions (elimination and supplementation) on ADHD.

**Methods:**

Our literature search resulted in 14 meta-analyses, six of which confined to double-blind placebo-controlled trials applying homogeneous diet interventions, i.e. artificial food color (AFC) elimination, a few-foods diet (FFD) and poly-unsaturated fatty acid (PUFA) supplementation. Effect sizes (ES) and Confidence intervals (CI) of study outcomes were depicted in a forest plot. I^2^ was calculated to assess heterogeneity if necessary and additional random effects subgroup meta-regression was conducted if substantial heterogeneity was present.

**Results:**

The AFC ESs were 0.44 (95% CI: 0.16–0.72, I^2^ = 11%) and 0.21 (95% CI: -0.02–0.43, I^2^ = 68%) [parent ratings], 0.08 (95% CI: -0.07–0.24, I^2^ = 0%) [teacher ratings] and 0.11 (95% CI: -0.13–0.34, I^2^ = 12%) [observer ratings]. The FFD ESs were 0.80 (95% CI: 0.41–1.19, I^2^ = 61%) [parent ratings] and 0.51 (95% CI: -0.02–1.04, I^2^ = 72%) [other ratings], while the PUFA ESs were 0.17 (95% CI: -0.03–0.38, I^2^ = 38%) [parent ratings], -0.05 (95% CI: -0.27–0.18, I^2^ = 0%) [teacher ratings] and 0.16 (95% CI: 0.01–0.31, I^2^ = 0%) [parent and teacher ratings]. Three meta-analyses (two FFD and one AFC) resulted in high I^2^ without presenting subgroup results. The FFD meta-analyses provided sufficient data to perform subgroup analyses on intervention type, resulting in a decrease of heterogeneity to 0% (diet design) and 37.8% (challenge design).

**Conclusion:**

Considering the small average ESs PUFA supplementation is unlikely to provide a tangible contribution to ADHD treatment, while further research is required for AFC elimination before advising this intervention as ADHD treatment. The average FFD ES is substantial, offering treatment opportunities in subgroups of children with ADHD not responding to or too young for medication. Further FFD research should focus on establishing the underlying mechanisms of food (e.g. incrimination of gut microbiota) to simplify the FFD approach in children with ADHD.

## Introduction

Attention-deficit/hyperactivity disorder (ADHD) is a child psychiatric disorder with a worldwide prevalence estimate of 6% [1] and characterized by impairing symptoms of inattention and/or hyperactivity and impulsive behavior, hampering the child’s development [2]. Children with ADHD are at risk for impaired academic performance [2], social isolation and peer problems [3], substance abuse [4], aggressive behavior and delinquency [5, 6]. In 50–65% of children with ADHD other psychiatric disorders like oppositional defiant disorder, conduct disorder and/or autism spectrum disorder are diagnosed as well [7–9], increasing the risk for adverse outcomes. Impairing symptoms of ADHD persist in up to 78% of children into adulthood [10]. Recent research has shown that suffering from ADHD may result in decreased life expectancy with more than double the risk of premature death from unnatural causes, like accidents, compared to people without ADHD [11]. In sum, ADHD seriously affects the quality of life of child, parents and siblings [3, 8], incurs high economic costs [12, 13] and is a long-term burden on families and society [14].

### Current ADHD therapy

The current multimodal standard of ADHD therapy consists of pharmacological treatment and/or behavioral or psycho-social therapy [15, 16]. Psychostimulants are first-choice pharmacological treatment [15] and have shown beneficial short-term efficacy, i.e. acute core symptom reduction [17, 18] in approximately 65–80% of children [19], a reduction of criminality rates [20] and of societal costs [21]. However, children taking psychostimulants may still meet the ADHD-criteria [22] and complete normalization of behavior is rare [23–25]. Furthermore, medication non-adherence occurs frequently [26, 27]: 30–50% of subjects stop taking medication within 12 months [28] and 66–80% within 3 years [17, 29, 30]. Apart from common side effects like sleep and appetite problems [14, 17], medication may also affect growth and long-term bone health [31]. Finally, drug treatment does not attenuate the increased risk for school dropout and unemployment [6]. In sum, better treatments preferentially aimed at prevention of ADHD in young children [22] and at targeting the underlying causes are welcome [14].

### ADHD etiology

Unfortunately, the causal pathways of ADHD are largely unknown; ADHD is a complex disorder and multiple factors may contribute to its etiology [32]. Apart from the involvement of many genes with a small effect [33], multiple pre-, peri-, and postnatal environmental factors may be risk factors for ADHD [34, 35]. To date, the synergistic action between genes and environment is generally acknowledged [36–38] and in ADHD genes ‘are thought to cause the disorder in the presence of unfavorable environmental conditions’ [33]. One of these conditions, though controversial [35], is diet [39–42].

Research into the effect of food on ADHD started forty years ago when pediatric allergist Benjamin Feingold hypothesized that both artificial food additives (colorings and flavors) and foods rich in salicylates (chemicals occurring naturally in some foods [43]) might be ‘important etiologic agents’ of the hyperkinetic syndrome [44]. The Feingold studies were followed by other elimination diet studies [45], investigating the effects of either artificial food color (AFC) elimination or of a diet eliminating many foods and additives, i.e. the few-foods diet (FFD), and by supplement studies investigating the effects of vitamins, minerals and poly-unsaturated fatty acids (PUFA) on ADHD [46].

### Recent reviews on ADHD and diet interventions

The efficacy of diet treatments in ADHD was recently evaluated in three reviews [40–42]. The main aim of reviews is to summarize the evidence on a specific topic, of which both researchers and clinicians may benefit [47]. However, the three reviews show divergent conclusions, i.e.: 1) there is evidence for a small effect of PUFA on ADHD, while the potential effect of AFC elimination remains unclear and more research is needed for a FFD [42]; 2) there is emerging consensus for the effect of food additives elimination (concurrently providing a food additive list to be given to a patient), while a one-week FFD is indicated in case of comorbid food allergy symptoms [41], and 3) none of the diet interventions are recommendable as ADHD treatment [40]. This divergence in conclusions might be explained by the fact that in previous reviews the results of uncontrolled and un-blinded studies [40], of studies amalgamating different types of diet interventions [40–42] and of meta-analyses not specifically aimed at children with ADHD or hyperactive behavior [41, 42] were included. Also, two reviews [41, 42] discussed studies [48, 49] as meta-analyses although the reported results were not derived from meta-analytic research, while none of three reviews [40–42] mentioned a study [50] that *was* a meta-analysis. The differences between the three previously published reviews and this review are listed in S1 Table.

This systematic review aims at determining the effect of diet interventions on the behavior of children with ADHD, based on published meta-analyses including double-blind placebo-controlled (DBPC) trials only and differentiating between types of interventions, thus addressing the above-mentioned limitations of previous reviews on ADHD and diet. To our knowledge this is the first review that exclusively focuses on meta-analyses of DBPC trials in children with ADHD, concomitantly segregating between the different types of diet intervention.

## Methods

No pre-specified protocol existed for this review. The Preferred Reporting Items for Systematic Reviews and Meta-analyses (PRISMA) guidelines were followed (see S1 Checklist). In December 2015 two researchers (LMP, KF) independently searched PubMed and Web of Science for diet meta-analyses without date limits or language restrictions, using the terms [(children or youth) AND (adhd or hyperactivity or hyperkinetic syndrome) AND (meta-analysis or Cochrane or systematic) AND (diet or food or nutrition or food colors or fatty acids) NOT (medication)]. Additionally, reference lists of the eligible meta-analyses and of recent reviews were scrutinized for further relevant meta-analyses. Meta-analyses were included if 1) they confined to studies with a DBPC design; 2) all studies were conducted in children meeting the criteria for ADHD or meeting the equivalent psychiatric standards relevant at the time the study was done (specifically in studies conducted previously to the introduction of the ADHD terminology), and 3) all studies applied one of the following interventions: either supplementation of one specific supplement (e.g. PUFA) or a group of supplements (e.g. vitamins and minerals), or elimination of one specific food or food component (e.g. sugar or AFC), some food groups (e.g. the Feingold diet or major allergens/gluten/high histamine) or many foods/food groups and additives (e.g. the FFD).

### Procedures

An inventory was made of type of intervention, study design, raters, outcome measures and effect size (ES) statistics reported in the identified meta-analyses. LMP and KF independently reviewed the meta-analyses, discussing discrepancies until consensus was reached. If different outcome measures were available, a measure was chosen that was most frequently used in the other meta-analyses in order to increase the homogeneity of results. If different ADHD symptom ratings were available, the total symptom score (i.e. inattention and hyperactivity/impulsivity) was included. If results were provided by different raters and reported for each rater separately, we included the ratings accordingly. Effect sizes (ES) and Confidence intervals (CI) of relevant study outcomes were depicted in a forest plot. An inventory was made of reported publication bias and of heterogeneity assessments. I^2^—as measure of heterogeneity—was calculated if necessary and possible. Substantial heterogeneity (I^2^ around 25% may be considered low, 50% moderate, and 75% high [51]) decreases the precision of the intervention’s effect [52] and the reliability of the results [51], underlining the importance to address the origin of heterogeneity [53]. Consequently, in case of substantial I^2^ without subgroup analytic results being provided, random effects subgroup meta-regression was conducted to assess the effect of subgrouping [52], using the original data provided in the studies included in the meta-analysis concerned.

### Search results

**Fig 1:**
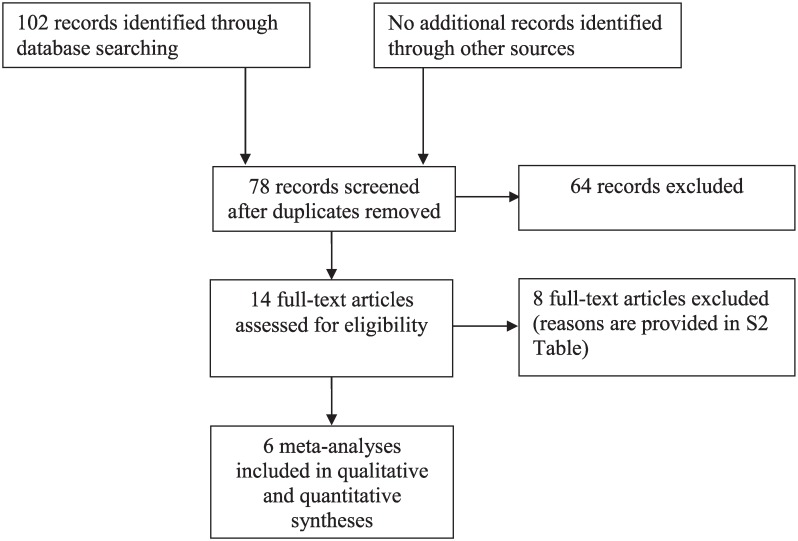
PRISMA* Flow diagram for the meta-analyses systematically reviewed. * PRISMA = Preferred Reporting Items for Systematic Reviews and Meta-analyses (www.prisma-statement.org).

The literature search, an overview of which is provided in Fig 1, resulted in fourteen meta-analyses, which were described in eleven different papers: six supplement meta-analyses—all investigating the effects of poly-unsaturated fatty acids (PUFA) [24, 54–58]—and eight elimination meta-analyses, examining respectively the effects of sugar [59], AFC [24, 60, 61], the Feingold diet [62], and the FFD [24, 50, 61] on ADHD (see S2 Table). No discrepancies between the researchers were found. One of eleven papers, describing three diet meta-analyses, applied unusual blinding criteria, i.e. ‘probably blinded’ assessments [24]. However, in all three meta-analyses [24] the assessments resulted from DBPC trials, thus being eligible to be included in this review. Eight of fourteen meta-analyses included studies in children not meeting the criteria for ADHD or hyperactivity, applying different types of diet or without a DBPC design. Six of fourteen meta-analyses met the inclusion criteria: two AFC [60, 61], two FFD [24, 50] and two PUFA [24, 54] meta-analyses.

## Results

The six eligible meta-analyses [24, 50, 54, 60, 61] are presented in Table 1. The interventions applied differed in composition, doses and duration. The AFC interventions mainly consisted of challenges with tartrazine or AFC mixtures and were given during 1–42 days [60], while the reported doses differed from 1 to 150 mg/day [60] or from 13 to 250 mg/day [61]. The PUFA challenges, given during four to 16 weeks [54], consisted of omega-3 PUFA, omega-6 PUFA, or a combination of both [24, 54], with doses ranging from 2.7 to 2800 mg/day [54], or from 120 to 2430 mg/day [24]. The FFD interventions consisted either of a FFD (during 9 days–4 weeks) or a challenge with specific foods (lasting 1–2 weeks) [24]. Benton reported that the FFD consisted of “lamb, chicken, potatoes, rice, banana, apple and brassica: foods chosen as they were unlikely to produce an adverse response”, but that the FFD might be adapted for each individual child [50].

**Table 1:** Description of the six meta-analyses included in this review.

*First author* *Publication year* (number of studies)	*Intervention* (number of children included in DBPC trial)	*Subject selection for diet responsiveness previous to inclusion*	*Study design* (DBPC diet, DBPC challenge, DBPC supplement)	*Rater*	*Outcome measure*^$^ *Conners’ rating scale*	*Results reported per rater*	*Publication bias*	*ES statistics* (ES positive, i.e. favors intervention)
**Schab** [60] 2004 (n = 15)	**AFC** (N = 136)	In 5/15 studies based on parent reports	Cross-over n = 15 (diet or challenge not specified)	P n = 13 T n = 6 O n = 4	P 10/13 T 6/6 O 2/4	Yes	Fail-safe N	SMD (P 10/13 Other 5/10)
**Nigg** [61] 2012 (n = 11*)	**AFC** (Not provided*)	Not provided*	Not provided*	P n = 11	Not provided*	Not provided*	Funnel plots Trim-and-fill	SMD (Not provided*)
**Benton** [50] 2007 (n = 5^)	**FFD** (N = 136)	In none of 5 studies	Cross-over n = 5 (diet 2/5 challenge 3/5)	P n = 4 O n = 1	P 4/4	No	Not reported	SMD (P 4/4 Other 1/1)
**Sonuga-Barke** [24] 2013 (n = 5^)^%^	**FFD** (N = 118)	In none of 5 studies	Cross-over n = 5 (diet 2/5 challenge 3/5)	P n = 1 T n = 1 DC n = 1 O n = 2	P 1/1 T 1/1 DC 1/1	No	Not reported**	SMD (P 1/1 Other 4/4)
**Gillies** [54] 2012 (n = 7)	**PUFA** (N = 762)	In 1/7 studies selection on PUFA deficiency symptoms [68]	Parallel n = 7 (supplement 7/7)^%%^	P n = 5 T n = 4 O n = 1	P 5/5 T 4/4	Yes	Not reported**	SMD (P 4/5 Other 3/5)
**Sonuga-Barke** [24] 2013 (n = 11)^%^	**PUFA** (N = 785)	In 1/11 studies selection on PUFA deficiency symptoms [68]	Cross-over n = 4 Parallel n = 7 (supplement 11/11)^%%^	P n = 4 T n = 6 P/T n = 1	P 2/4 T 6/6	No	Not reported**	SMD (P 4/4 Other 4/7)

AFC = artificial food colors; FFD = few-foods diet; PUFA = poly-unsaturated fatty acids; DBPC = double-blind placebo-controlled; P = parent; T = teacher; DC = day-care; O = observer; P/T = combined parent and teacher ratings; Other = all raters except parents. ES = effect size.

*Nigg et al. included 20 studies. 11/20 studies concerned hyperactive children only, the parent ratings of which are provided in Nigg et al.’s Table 2 [61]. Numbers of children included, design, results per rater and outcome measures are not provided for the 11 studies.

^The two FFD meta-analyses, including the same five FFD studies, reported the results of different raters.

^%^Probably blinded assessments’ meta-analysis.

^%%^In one study more than half of the children in the PUFA-group also received a multivitamin supplement [69].

^$^Missing numbers of raters in this column used a variety of other rating scales.

**Publication bias was not reported due to the small numbers of trials.

**Fig 2:**
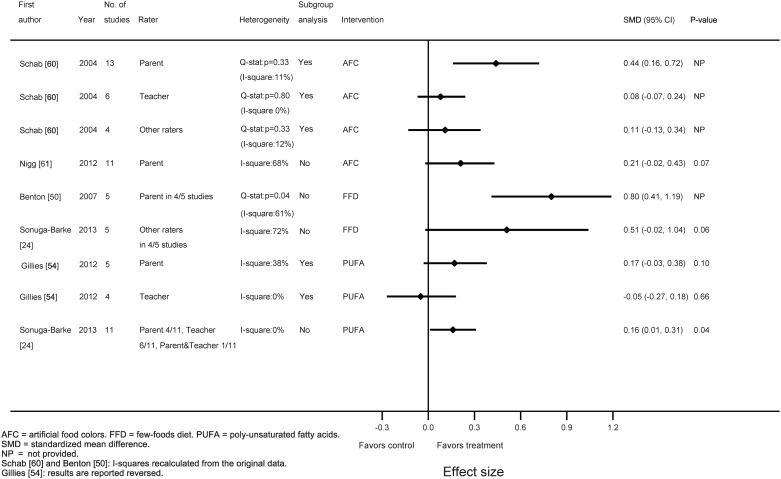
Characteristics and outcomes of the six diet meta-analyses included in this systematic review. All meta-analyses included DBPC trials only, conducted in children meeting the criteria of ADHD.

Two meta-analyses evaluating either AFC elimination [61] or PUFA supplementation [24] referred to the previously conducted meta-analyses [54, 60], while the FFD meta-analysis [24] did not mention the former FFD meta-analysis [50] (see S2 Table). One of six meta-analyses differentiated between outcome measures (e.g. ADHD total symptoms, ADHD inattention, ADHD hyperactivity) and study design (e.g. parallel, cross-over, blinded challenge, blinded diet) [54]: concerning this meta-analysis we report the total ADHD symptoms’ results ensuing from the parallel studies’ meta-analyses, covering seven of nine studies [54]. In all meta-analyses standardized mean differences (SMD) were used as effect sizes (see S1 Data part 1); standardized ESs of 0.2 are considered to correspond to a small effect, 0.5 to a medium and 0.8 to a large effect [63]. The AFC ESs were 0.44 (95% CI: 0.16–0.72, I^2^ = 11%) and 0.21 (95% CI: -0.02–0.43, p = 0.07, I^2^ = 68%) [parent ratings], 0.08 (95% CI: -0.07–0.24, I^2^ = 0%) [teacher ratings] and 0.11 (95% CI: -0.13–0.34, I^2^ = 12%) [observer ratings]. The FFD ESs were 0.80 (95% CI: 0.41–1.19, I^2^ = 61%) [parent ratings] and 0.51 (95% CI: -0.02–1.04, p = 0.06, I^2^ = 72%) [other ratings], while the PUFA ESs were 0.17 (95% CI: -0.03–0.38, p = 0.10, I^2^ = 38%) [parent ratings], -0.05 (95% CI: -0.27–0.18, p = 0.66, I^2^ = 0%) [teacher ratings] and 0.16 (95% CI: 0.01–0.31, p = 0.04, I^2^ = 0%) [parent and teacher ratings]. The results are depicted in Fig 2.

In two of six meta-analyses heterogeneity was assessed by means of Q-statistics, a method generally applied in meta-analyses published previous to 2009 [50, 60, 64–67]. We calculated I^2^ using the Q statistics or the raw data provided (see Fig 2). Three of six meta-analyses (one AFC [60] and two PUFA [24, 54]) showed I^2^ values less than 40%, while three meta-analyses (one AFC [61] and two FFD [24, 50]) reported substantial I^2^ values without presenting subgroup results. Of the latter meta-analyses we performed sub-analyses to investigate the effect of intervention and rater on heterogeneity. However, since Nigg et al. [61] did not report which studies were incorporated in the AFC meta-analysis of eleven studies including hyperactive children only, thus prohibiting further sub-analytic calculations, only the sub-analytic results of the two FFD meta-analyses [24, 50] are reported below.

### Sub-analysis based on the FFD meta-analysis by Benton

**Fig 3:**
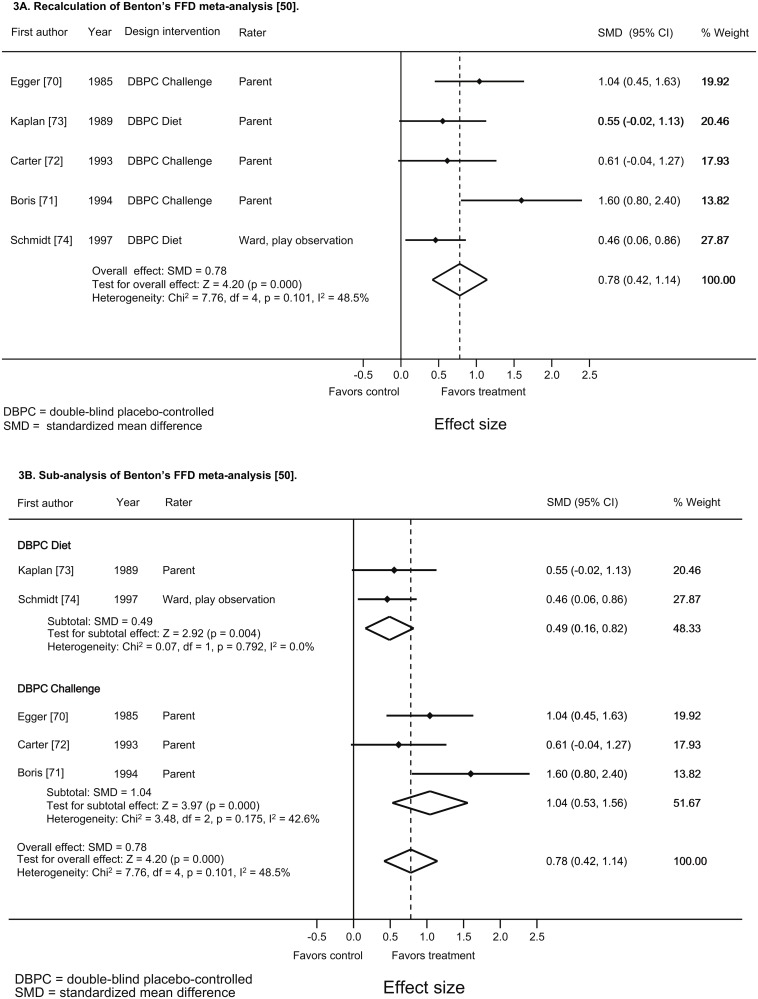
Recalculation and sub-analysis of Benton’s FFD meta-analysis [50] (3A and 3B) based on the data derived from the original articles. Forest plot of FFD effects and homogeneity statistics.

The FFD meta-analysis by Benton [50], published in 2007, resulted in I^2^ = 61%, which is considerable. Subgroup analytic results were not provided; consequently we performed sub-analyses to assess the effect of subgrouping. However, we first performed a recalculation of this meta-analysis based on the data in the original papers to verify the results, since in one study included by Benton [50] mean and SD were provided in a figure only [70], while in another study [71] the number of subjects (n = 16) differed from the number provided by Benton (n = 26) [50]. Please see S1 Data, part 2, for the procedure followed. The results of this recalculation are reported in Fig 3A; the data derived from the original articles and used to perform the recalculation are presented in S3 Table.

Benton included the DBPC parent ratings resulting from four of five RCTs [70–73]. The fifth study, by Schmidt et al. [74] did not provide parent ratings, since this RCT was an inpatient study reporting three different outcomes: specialized teacher ratings, ward observation ratings and test observation results. Benton included the ward observation measurements, which in an in-patient population would come the closest to parent measurements. Considering the homogeneity of raters we performed a random-effects meta-regression to assess the effect of intervention type (i.e. diet or challenge) on heterogeneity only. The outcomes of the sub-analyses are shown in Fig 3B, resulting in a decrease of the heterogeneity in the subgroups compared to the overall analysis.

### Sub-analysis based on the FFD meta-analysis by Sonuga-Barke et al.

The FFD meta-analysis by Sonuga-Barke et al. (see page 283, Fig 3A [24]), published in 2013, also resulted in considerable heterogeneity (I^2^ = 72%) without providing subgroup-analyses. Preparatory to performing sub-analyses we executed a recalculation of this meta-analysis based on the data derived from the original papers (see S3 Table), since Sonuga-Barke et al. [24] neither reported the numbers of subjects included in the DBPC assessments nor means and SDs necessary to calculate ES and CI.

Contrary to Benton [50], including DBPC parent ratings, Sonuga-Barke et al. [24] predominantly focused on DBPC other raters’ results. Although acknowledging that in DBPC trials both parent and teacher ratings are ‘probably blinded’ [24], direct observations or teacher ratings (in that order of preference) were considered ‘better probably blinded’ [24]. If available, these ratings (provided by teachers [74], day-care workers [73] and psychologists [70, 72]) were included by Sonuga-Barke et al. [24]; if not available, the DBPC parent ratings were included [71]. We intended to use the same measurements as Sonuga-Barke et al. [24] in our recalculation. However, we were compelled to make different choices concerning two [70, 74] of five studies, and noted an important difference in a third study [71]:

1. In the study by Egger et al. parent and psychologist ratings were provided [70]. Sonuga-Barke et al. included the psychologist’s ratings, because the parent results were presented in graphical form with no SDs (see Sonuga-Barke et al.’s supplementary appendix page 23) [24]. We were unable to include the psychologist’s ratings in our recalculation since Egger et al. [70] only provided mean and the paired t-value but not the correlation (r), which is needed to calculate the ES from a paired t-value. However, we were able to estimate ES and 95% CI of the parental data based on the graphical representation of the data (using Microsoft Publisher ‘s ruler). Consequently, contrary to Sonuga-Barke et al. [24] we included Egger et al.’s [70] parent ratings results.
2. In the study by Schmidt et al., conducted in an inpatient population, teacher ratings and two observer ratings (ward and test observations) were provided [74]. Sonuga-Barke et al. [24] included the teacher measurements in their meta-analysis, while we included the test observation ratings, for two reasons. First, Schmidt et al. [74] reported that, due to the specialist setting in their clinic school with highly experienced teachers and only one to three children per teacher, the teacher ratings neither revealed behavioral problems at the start of the trial nor established the beneficial effect of medication. Commensurately, biased teacher results were obtained in a laboratory school study evaluating the effect of medication, the teachers being unable to differentiate between children taking medication or placebo, probably due to the therapeutically beneficial effect of both good structure and small classes [75]. Taking the equally specialized setting into consideration, Schmidt et al. [74] excluded the teacher ratings’ results from further analysis, which is in line with reviews on other ADHD treatments, excluding results from laboratory school studies from evaluation as well [76–78]. Second, including teacher ratings would not be in accordance with Sonuga-Barke et al.’s statement that in home-based treatments (in Schmidt et al.’s study [74] the ward being the children’s temporary home) observer ratings should prevail over teacher ratings [24]. Based on these reasons we included the ‘best probably blinded’ test observation results in our recalculation, since ward play observations in an inpatient population would be comparable to parent observations.
3. Sonuga-Barke et al. [24] presented an incorrect ES concerning Boris & Mandel’s study, given the means and SD’s provided in the original paper [71]. Our recalculation includes the original data provided by Boris & Mandel [71].

**Fig 4:**
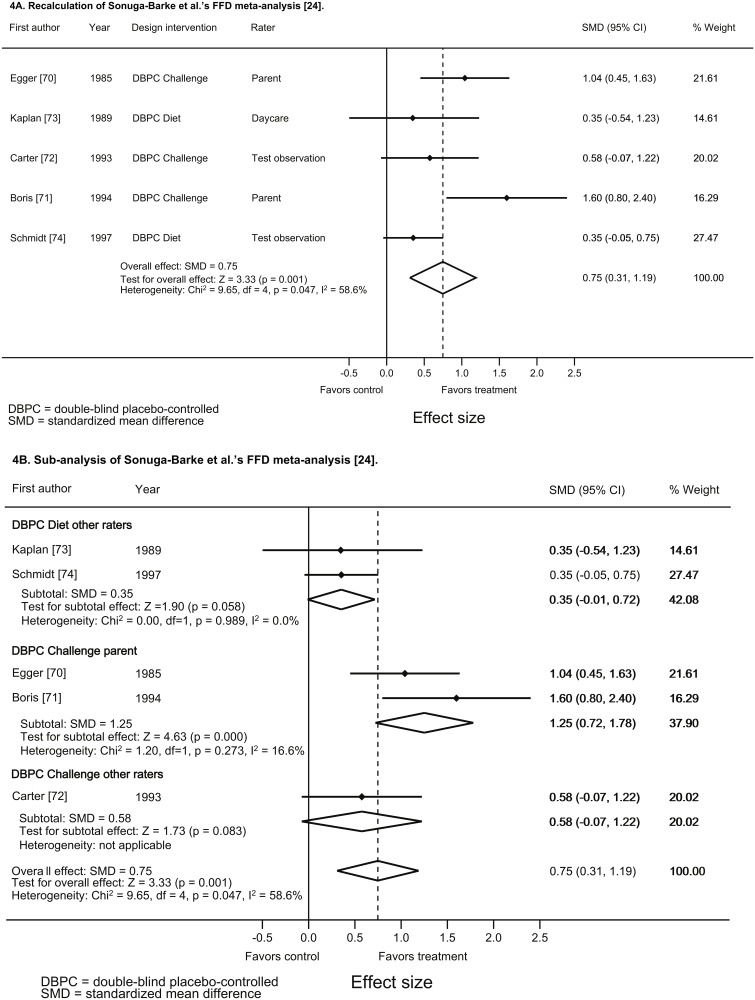
Recalculation and sub-analysis of Sonuga-Barke et al.’s FFD meta-analysis [24] (4A and 4B) based on the data derived from the original articles. Forest plot of FFD effects and homogeneity statistics.

The meta-analytic results of our recalculation are provided in Fig 4A, commensurate to the results by Sonuga-Barke et al. [24] based on the ‘best probably blinded’ ratings and showing considerable heterogeneity. Additionally, to approach the meta-analysis by Sonuga-Barke et al. [24] as closest as possible we also performed a recalculation including Egger et al.’s psychologist ratings [70] based on the figures provided by Sonuga-Barke et al. [24] in their Fig 3A, page 283, the results of which are presented in S1 Fig. Finally, we performed a random effects meta-regression to assess the effect of intervention type (i.e. diet or challenge) and of raters [i.e. parents [70, 71] and other raters (i.e. day-care [73], psychologist [72] and test observers [74])] on heterogeneity (see Fig 4B): the results show that subgrouping decreases heterogeneity in the subgroups compared to the overall analysis.

In sum, both FFD meta-analyses [24, 50] provide important though different and complementary information: one focusing on DBPC parent ratings [50], the other predominantly including other raters’ assessments [24]. To understand the real merits of an intervention a complete picture is needed rather than limited coverages of outcomes, since meta-analyses not providing all core outcomes are considered suboptimal [79]. Consequently, a complete overview of forest plots and homogeneity statistics, including all DBPC FFD ratings provided in Figs 3 and 4, is depicted in S2 Fig.

### Risk of bias

**Fig 5:**
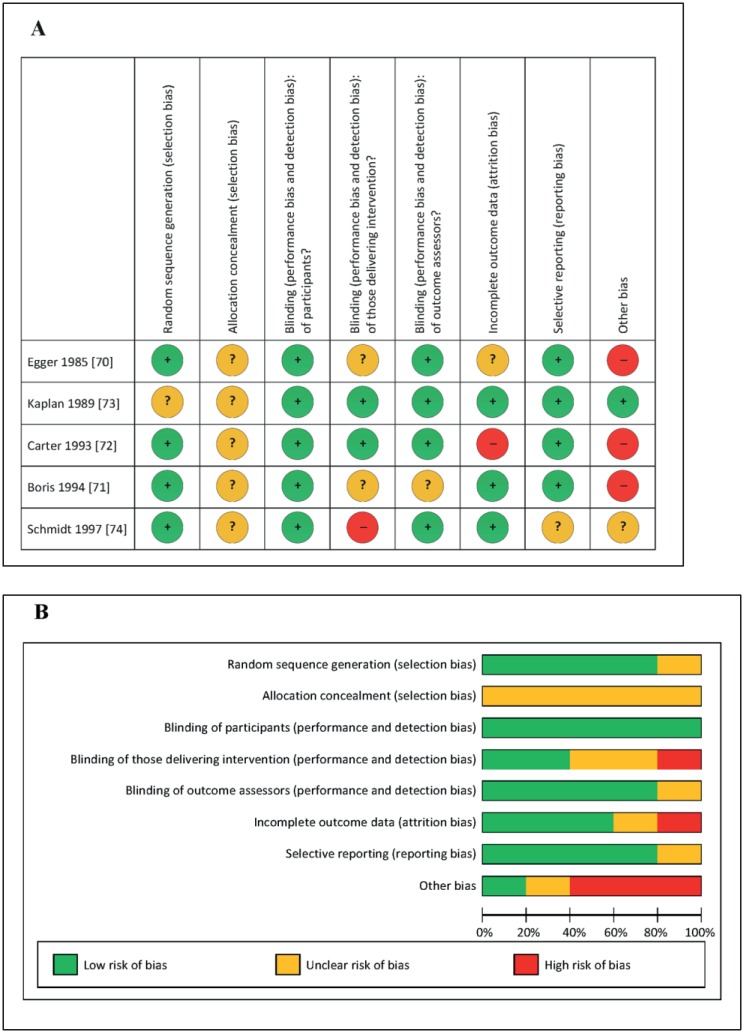
Risk of bias graphs: review authors’ judgements about each risk of bias item. (A) Bias presented for each individual study. (B) Bias presented as percentages across all included studies.

We additionally evaluated the risk of bias. Commensurate to Sonuga-Barke et al. [24] we were unable to assess publication bias by means of funnel plots, since it is recommended that at least ten studies are needed for funnel plots to be reliable [80]. However, LMP and KF independently assessed the risk of bias of each trial included in Figs 3 and 4 following the guidelines provided in the Cochrane handbook for systematic reviews of intervention, version 5.1.0. [52]; disagreements were dissolved by RRP. The results are presented in Fig 5.

## Discussion

The results of this systematic review, conducted to synthesize meta-analytic results of diet interventions in children with ADHD in order to determine the effectiveness of diet treatments on ADHD, show that the average ESs of AFC elimination and PUFA supplementation are too small to contribute significantly to ADHD treatment, while the FFD ESs are medium to large, possibly offering novel treatment opportunities. The differences in outcomes between diet types, the quality of the evidence provided in the subgroup meta-analyses, the differences in conclusions between our and previous reviews, and the importance of addressing CIs and heterogeneity is discussed below.

### Differences in outcomes between diet types

Fig 2 shows that the FFD ESs are considerably larger than the ESs of AFC and PUFA, which might be explained by inadequate blinding of the FFD; an intervention eliminating many foods will conceivably be less easier to blind than AFC and PUFA, which can be hidden in cookies, candy bars or capsules. However, this drawback was obviated in all DBPC FFD studies by drastically adapting the intervention, thus securing the blinding (see S1 Text). Indeed, Sonuga-Barke et al. reported that 5/5 FFD studies and 8/11 PUFA studies included in their meta-analyses of probably blinded measurements received the maximum JADAD score for blinding [24], indicating that the blinding method was both described and appropriate, and that neither participants nor assessors (e.g. parents, teachers, other raters) were able to identify the intervention applied [81].

The higher FFD ESs might also be the consequence of parental investments necessary to apply the intervention, specifically since the FFD is considered a strenuous intervention [48, 70, 72, 74, 82, 83]. However, in adequately conducted DBPC trials parental investments are deemed commensurable in verum and placebo groups. Furthermore, parental investments in the five DBPC FFD studies were marginal: one FFD study was an inpatient study and no parents were involved [74], in the second FFD study ready-made meals were supplied [73], and in the three FFD challenge studies the diet impact was limited to the challenges provided by the research team [70–72]. Consequently, parental investments are unlikely to underlie the high FFD ESs.

The disparity in ESs might also be explained by the numbers of foods involved. According to Rucklidge & Kaplan it would be unlikely that one supplemented nutrient resolved all vulnerabilities present in a complex disorder like ADHD, thus explicating the small behavioral effects of supplement research that focuses on single nutrients [84]. Comparably, Benton argued that the effect of an additives-free diet might be hidden completely by adverse effects to other foods still in the diet, because potentially many foods may trigger adverse behavioral effects, thus highlighting the importance in diet research to focus on many foods [50]. This line of reasoning is corroborated by the results of the FFD studies, showing that large numbers of foods as well as individual response differences are involved in behavioral changes [70, 72], underlining that more restricted diet interventions may result in larger behavioral effects [71].

Finally, low ESs may be explained by suboptimal intervention conditions. Contrary to most DBPC medication studies, using optimal medication doses because suboptimal doses would result in biased and less optimal outcomes [78], in most DBPC diet studies suboptimal conditions were noted. In the AFC meta-analyses differences in AFC composition, dose, duration of exposure, washout period and the timespan between ingestion and testing were reported; these dissimilarities might act as confounding factors, resulting in underestimation and high variability of results [60, 61]. Commensurately, as reported by Gillies et al., the DBPC PUFA studies differed in dosage and type of fatty acids (either omega-3, omega-6, or a combination of both) and in duration of supplementation [54], while the suboptimal diet applied in the FFD studies (see S1 Text) may also have affected the FFD results.

### Differences in conclusions between our and previous reviews

Unlike the conclusions of previous reviews on ADHD and diet [40–42], our review suggests that there is convincing evidence for the effect of a FFD on ADHD. The difference in conclusions may be explained as follows: First, in accordance with the recommendation of the American Psychological Association to base discussion and interpretation of results on ES and CI [85], our conclusions are based on ESs rather than on p-values. Study conclusions based on p-values only may not accurately represent the clinical relevance of an intervention [86–89]: p-values primarily provide information on the statistical (non-) significance and are highly dependent of sample sizes, i.e. small changes in sample size may convert the statistical outcomes from insignificant to significant or vice versa [63, 86, 90]. Conversely, ESs provide clinically relevant information and are hardly affected by changes in sample size [63, 86, 90]. Given that sample sizes in pediatric research frequently are small, studies may show statistically non-significant differences (p-values > 0.05) even when the ESs are large [86], elucidating that statistical non-significance is not equivalent to clinical irrelevance [63, 91]. However, although ES (and CI, the importance of which will be discussed below) is considered important to assess the average clinical relevance of an intervention [52], in medical research p-values are still often used as the decisive information to accept or reject study outcomes, illustrated by two meta-analyses included in this review, either resulting in significant p-values and small ESs (PUFA ES = 0.16; p = 0.04) [24] or in non-significant p-values and medium ESs (FFD ES = 0.51; p = 0.06) [24]. Based on these results it was concluded that PUFA supplementation showed beneficial, though small, effects on ADHD, while further evidence for efficacy was required for a FFD [24].

In addition, the impact of statistical significance in medical research may also be deduced from the PUFA meta-analyses included in this review. The first study reported statistically non-significant results (ES = 0.17), concluding that “Overall, there is little evidence that PUFA supplementation provides any benefit for the symptoms of ADHD in children” [54]. Conversely, the second study reported statistically significant results (ES = 0.16), concluding that PUFA supplementation “produced small but significant reductions in ADHD symptoms even with probably blinded assessments” [24].

The confusion concerning statistical significance and clinical relevance in medical research is demonstrated in one of the recent reviews on diet and ADHD [40], stating that the FFD resulted in ‘an insignificant effect when looking only at assessments made by an independent blinded assessor’, the words ‘insignificant effect’ pointing at the medium ES of 0.51 and the insignificant p-value of 0.06 [24]. Furthermore, the misleading inference that may result from meta-analytic interpretations predominantly based on p-values is elucidated in S1 Fig, including the same rating results as reported by Sonuga-Barke et al. [24], except for Schmidt et al.’s teacher ratings results, which were replaced by the test observation rating results [74]. The ES calculated in S1 Fig (ES = 0.57) is comparable to the ES calculated by Sonuga-Barke et al. (ES = 0.51 [24]). However, the statistical insignificance (p = 0.06) found by Sonuga-Barke et al. [24] becomes statistically significant (p = 0.024). It might be conceivable that inclusion of Schmidt et al.’s test observation ratings rather than the teacher ratings [74] by Sonuga-Barke et al. [24] would have affected their conclusions and subsequently those of recent reviews [40–42] and keynote papers [92, 93].

Second, the three recent reviews [40–42] did not discuss or refer to the previously published FFD meta-analysis by Benton [50]. Our review is the first review interpreting the FFD results in the context of previous research, i.e. including the results of the first FFD meta-analysis [50] as well, which, according to the Scottish ADHD guidelines, provides the highest level of evidence (1++), indicating that it is a high-quality meta-analysis with a very low risk of bias [94]. Interpretation of meta-analytic results in the context of other evidence is considered important [85–87, 95, 96]. Indeed, according to Helfer et al. ‘journals should make the discussion of related meta-analyses mandatory’ to improve the transparency and value of meta-analyses and to enhance evidence-based practice [97].

### The importance of addressing CI and I^2^

When evaluating the clinical relevance of an intervention not only the average effect of an intervention, i.e. the ES, but also the range of the average treatment effect, i.e. the 95% CI, should be considered [98], taking into account that the width of CIs, like p-values, is affected by the sample size: the smaller the sample, the wider the CI [63, 88, 99]. In addition, the 95% CI width depends on the standard deviation (SD): the wider the SD, the wider the CI [88, 99]. Wide SDs may result from a wide distribution of post-intervention scores in the treatment group, ensuing from population variability and individual response differences—some subjects showing large effects at post treatment, others showing small or no effects [100]. Large individual differences in response might occur in FFD studies [70, 74], offering an explanation for the wider FFD 95% CI’s when compared to the 95% CI’s of AFC and PUFA. Consequently, interpretation of the width of 95% CI should always be done in light of sample size and SD.

Furthermore, in meta-analytic research heterogeneity testing is important to estimate the consistency of study outcomes. Specifically in meta-analyses combining different raters and interventions heterogeneity is to be expected [51]; a meaningful meta-analytic summary can only be provided when the data included are more or less homogeneous [52], which can be achieved by means of sub-analyses, thus increasing the reliability of the findings [51]. Fig 4B, providing an overview of FFD sub-analyses, shows that the overall I^2^ of 58.6% decreases to 0% (diet design, other raters) and 16.6% (challenge design, parent ratings) when subgrouping diet types and raters. This reduction is comparable to the decrease of I^2^ following subgrouping in medication meta-analyses [78].

However, despite a decrease of heterogeneity and the concurrent increase of consistency and reliability of the beneficial effect of a FFD on ADHD in groups of children, subgroup meta-analyses do not provide information of whether an intervention would be beneficial for an individual patient, neither do high ESs and small 95% CI’s, representing the average treatment effect only [98]. For example, drug meta-analyses often show impressive ESs with narrow 95% CIs not including zero (i.e. statistically significant) [64, 77], although the heterogeneous response to medication—some children responding well while others not responding at all or responding sub-optimal—is well established [75, 101]. Indeed, since ‘ADHD is a heterogeneous disorder with multiple causes that probably differ between individuals’ [92], and interpersonal variability may be high when diet is concerned [102], differentiation between responders and non-responders and determination of response predictors [41] are important to establish the clinical importance of an intervention for each individual child.

### Quality of the evidence provided in the subgroup analyses

The bias results concerning blinding and attrition, presented in Fig 5, are commensurable with the trial quality ratings of the 5 DBPC FFD studies reported by Sonuga-Barke et al. [24] who used the Jadad scale, providing scores ranging from 0–5 (for randomization (0–2 points), blinding (0–2 points) and attrition (0–1 point) [81]). The FFD studies received Jadad scores of 3 [73], 4 [74] and 5 points [70–72], i.e. all were rated fair or above [24]. Two items reported in Fig 5, i.e. ‘allocation concealment’ and ‘other bias’, need to be addressed here. First, although all studies reported randomization, none described the method applied to conceal the allocation, which is important to prevent ‘foreknowledge of intervention assignment’ [52]. According to Mills et al. allocation concealment is hardly reported in crossover trials [103], perhaps because all participants automatically receive both treatments, thus prohibiting selective enrolment based on expectations. Second, in three of five studies one of the potential sources of other bias listed in the Cochrane handbook for systematic reviews of interventions was noted, i.e. ‘pre-randomization administration of an intervention that could enhance or diminish the effect of a subsequent, randomized, intervention’ [52]. However, responsiveness selection previous to randomization was not only applied in FFD studies using a challenge design [70–72] (see S1 Text), but also in AFC studies [60] and medication studies [64]. Indeed, a recent Cochrane review, evaluating the effect of methylphenidate on ADHD, reported that cohort selection and exclusion of placebo-responders as well as exclusion of non-responders to methylphenidate often occur in medication trials [104].

Furthermore, S2 Fig shows that DBPC observer ratings resulted in lower ESs than DBPC parent measurements. The divergence in results between raters can be explained by the fact that in different settings different aspects of the child’s behavior are observed, each rater providing ‘different perspectives on therapeutic effects’ [105]. For example, in the Multimodal Treatment of ADHD (MTA) study [106], investigating the effects of both behavioral and pharmacological treatment in children with ADHD, the blinded observer measurements did not show significant treatment effects on the child’s behavior [107], contrary to the parent ratings [106]. Indeed, an observational setting may lack ‘ecological validity, as these ratings are based on only a snapshot of the child’s behavior’ [24] and the behavioral symptoms may not be present in different or new situations [108]. According to Sonuga-Barke et al. neither laboratory observer ratings nor parent ratings can be considered better measures of treatment effect, since each provides different information [105]. In fact, drug treatment effects in children with ADHD are usually assessed using parent ratings, which is considered an ‘ecologically valid method of assessment’ [105].

Based on the results depicted in Figs 3, 4 and S2 we hypothesize that the results of the FFD studies using a diet design [73, 74] could be applicable to the general population of children with ADHD, provided that parents are interested in diet treatment, while the results of the challenge studies [70–72] would be applicable to children with ADHD who are alleged to respond to foods. However, the FFD challenge studies have an important additional merit: the open parental findings obtained previously to randomization were confirmed in a DBPC setting in each of the challenge studies [70–72], thus providing evidence for the reliability of open rating results in FFD studies. Consequently, the results of the challenge studies might be extrapolated to the general population of children with ADHD as well, taking into account that many subjects participating had physical symptoms (though those without did as well as those with [70]) or had parents that were specifically interested in diet treatment [72]. Finally, the challenge study results show that all kinds of foods may provoke ADHD behavior in children, underlining the importance of applying a diet as restricted as possible to establish the effect of food on ADHD.

## Limitations

This review has some limitations. First, we limited our search to PubMed and Web of Science, so we may have missed relevant meta-analyses, although we also searched reference lists of all 14 meta-analyses and of recent reviews on the topic. Second, our review was limited to meta-analytic reviews; other reviews were not included. Third, we only included published meta-analyses that focused on DBPC trials investigating the effect of diet on the behavior of children meeting the criteria for ADHD or the equivalent psychiatric standards relevant at the time the study was done. Fourth, it is conceivable that only parents interested in diet treatment will participate in a diet trial, thus limiting the results of this review to children whose parents are receptive to a dietary approach of ADHD.

## Clinical implications and future research

Our systematic review, evaluating the results of all published meta-analyses including DBPC trials investigating the effect of diet interventions on ADHD, shows that the average ESs are -0.05 to 0.17 (PUFA), 0.08 to 0.44 (AFC) and 0.51 to 0.80 (FFD). First, the PUFA ESs are small to negligible, warranting the conclusion that as yet PUFA supplementation should not be advised as a treatment of ADHD, although it should be acknowledged that the individual effect of an intervention may be different from the average group effect. When searching www.isrctn.com and www.ClinicalTrials.gov (search date April 2016) for registered and on-going PUFA, AFC and FFD trials in ADHD (key words: diet, food, nutrition) we found 26 registered clinical PUFA trials, 7 of which on-going, illustrating that the interest in PUFA research is substantial. Further PUFA research might 1) address the limitations reported by Gillies et al. [54], 2) include blood tests to establish any PUFA deficiencies [58] and, in light of the increasing evidence for omega-6 and omega-3 PUFA competition for common enzymes [109], 3) focus on the quantity of omega-3 and omega-6 PUFA already present in the child’s diet [110].

Second, the AFC ESs, though exceeding the PUFA ESs, are too small to contribute to ADHD treatment. Consequently, provision of additives lists that can be given to parents of children with ADHD [41] is not warranted yet. Still, the AFC ESs are too large to dismiss. Since we did not find any registered or on-going AFC trials we suggest future research into the effect of AFCs on ADHD to be incorporated in further FFD research, i.e. children responding to a FFD should receive appropriate challenges with AFCs according to the advice given by Schab & Trinh [60].

Third, the FFD ESs are medium to large. Combined with the decrease of heterogeneity resulting from subgroup-analyses, these results would justify administration of this intervention in children with ADHD, in line with a previous implementation advice [111]. However, contrary to medication a FFD is not a long-term treatment, but a short-term diagnostic procedure, appropriately described by Rytter et al.: ‘Few Foods Diets are not meant as treatment, but only as a method to identify diet-sensitive children. The actual treatment is the individually tailored diet designed after repeated challenges have identified which food items should be avoided’ [40]. Research has shown that this ‘few-foods approach’—i.e. a short-term FFD followed by food challenges in children showing clinically relevant behavioral improvements (diet responders), eventually resulting in a personalized diet advice—would be achievable but may take at least one year [70, 72], is considered burdensome [48, 70, 72, 74, 82, 83], is feasible only in motivated families with good family structure [112] and would be easier to apply in younger children [113]. Hence, large-scale implementation of the few-foods approach would not be a realistic recommendation. Until further research into the mechanism of food in children with ADHD results in easier methods to define whether or not a child reacts to food—and if so, to which foods—implementation of the few-foods approach should solely be considered in children not responding to medication and in young children with ADHD in whom medication should be applied with caution [114].

When searching for registered and ongoing FFD trials we only found two RCTs, both already published [48, 82]. In light of the evidence available, further FFD research is important and should move beyond the question of whether a FFD *may* affect ADHD towards the question *how* food exerts its effect, and in which children. Establishing the biological basis of environmental influences on psychiatric disorders, including research into neuroendocrine mechanisms, is important to define ‘how environments get under the skin’ [115]. In the specific case of the FFD it is of vital importance to facilitate or even supersede the few-foods approach, which is very aggravating and is unlikely to become a generally applicable procedure in children with ADHD. Further research might focus on the gut-brain axis, the gut microbiota and their metabolites, and the enteric nervous system. During the last decade, gut-brain signaling research has shown that the microbiota (i.e. the myriads of microorganisms colonizing the digestive tract) and its microbiome (i.e. the collective microbiota genes) may modulate behavior [116]. In fact, the gut microbiota responds rapidly to a change of diet [117, 118] and produces neurochemicals comparable to the neurochemicals produced by the brain [119, 120]. Further research may 1) result in finding biomarkers or pathways, e.g. alterations in metabolites that are regulated by the gut flora [121], differences in neurotransmitters or in microbiota composition; 2) provide an explanation for individual differences in diet response and the associated wide SD’s and CIs, concurrently offering the opportunity to differentiate between types of ADHD, and 3) offer novel diagnostic and treatment possibilities (e.g. specific probiotics) for children with ADHD. In light of the high frequency of comorbidity in ADHD [122] and the recently established shared genetic etiology and pathophysiology with major psychiatric disorders [123], these findings may be of importance to other psychiatric disorders as well.

## Recommendations

Several recommendations ensue from this review. First, we noted heterogeneities in nomenclature of diet and of blinding. Recommendations for unequivocal diet and blinding nomenclatures are given in S2 and S3 Texts. Second, in accordance with the recent advice by Fabiano et al. not to combine diverse psychosocial intervention results into one aggregate analysis [124], we suggest that future studies on ADHD and diet segregate between different diet types. Third, although a DBPC design is the gold standard, some interventions, like behavioral therapy [106, 125, 126] and an optimal FFD (see S1 Text), are difficult to blind. Given the advice that blinded designs in future intervention studies should ‘not compromise the quality of the treatment being evaluated’ [24] we suggest further FFD research to apply single-blinded ratings administering a non-disclosure procedure to complement parent and teacher ratings, a method often used in studies difficult to blind [48, 125–128]. Fourth, when applying a FFD in practice the most optimal intervention should be applied, which is consistent with medication guidelines, advising dose titration to achieve maximum benefit with minimum adverse effects [129]. Fifth, we suggest future RCTs to include information about the frequency of comorbidity and about the intervention’s effect on comorbid disorders. Finally, the importance of scrutiny when including rating results in meta-analytic calculations, of performing sub-analytic calculations in case of high I^2^ and of providing a complete picture of ratings cannot be underestimated in meta-analytic research.

## Conclusions

Based on double-blind placebo-controlled evidence our systematic review shows that the effect sizes of AFC-free diets (small to medium) and PUFA supplementation (negligible to small) warrant the conclusion that as yet these interventions should not be advised as general ADHD treatment. Conversely, the effect sizes of a few-foods diet are medium to large, justifying implementation of a diagnostic FFD in subgroups of children with ADHD, thus offering innovative treatment opportunities for ADHD. Further FFD research should focus on the mechanism of food in children with ADHD. Finding pathways may result in an easier diagnostic procedure to differentiate between diet responders and nonresponders, in an easier therapeutic approach in children responding to foods and in a personalized disease strategy. The findings of this review are in line with the position of the International Society for Nutritional Psychiatry Research, recently stating that there is ‘emerging and compelling evidence for nutrition as a crucial factor’ in mental disorders, and suggesting that ‘consideration of nutrition should be part of standard practice’ [130].

## Supporting Information

S1 PRISMA Checklist (PDF)

S1 Data Overview of effect size calculations. Part 1. Effect size calculations in the meta-analyses included in this review (see Fig 2). Part 2. Effect size calculations applied in our recalculations and subgroup meta-analyses (see Figs 3, 4 and S2).
(PDF)

S1 Table Characteristics of the most recently published reviews discussing the effect of diet on ADHD, and of the current review. (PDF)

S2 Table Summary of characteristics of 14 meta-analyses evaluating diet interventions in ADHD, including reasons for exclusion (depicted by ‘No’). (PDF)

S3 Table Overview of FFD RCT’s raters, design, and of the data provided in the articles (either graphical or in figures) and included in Figs 3, 4 and S2 (denoted by ‘1’). (PDF)

S1 Fig Data provided in the probably blinded assessments’ FFD meta-analysis by Sonuga-Barke et al. (see Sonuga-Barke et al.’s page 283, Fig 3A), except for the teacher data provided by Schmidt et al., which are replaced by the test observation data. Forest plot of FFD effects and homogeneity statistics.
(PDF)

S2 Fig Sub-analysis of DBPC RCTs applying a FFD intervention, subgrouped per rater (parent or other). Forest plot of FFD effects and homogeneity statistics.
(PDF)

S1 Text Strengths and weaknesses of double-blind placebo-controlled RCTs investigating the effect of a few-foods diet on ADHD. (PDF)

S2 Text Proposition for an unambiguous nomenclature of diet research. (PDF)

S3 Text Proposition for unambiguous definitions of blinding. (PDF)
