# Supplementary material for: Diet and ADHD, Reviewing the Evidence: A Systematic Review of Meta-Analyses of Double-Blind Placebo-Controlled Trials Evaluating the Efficacy of Diet Interventions on the Behavior of Children with ADHD
Source: PLoS One. 2017 Jan 25;12(1):e0169277. doi: 10.1371/journal.pone.0169277 (PMC5266211; doi:10.1371/journal.pone.0169277)
Supplement: S1 Data — Part 1. Effect size calculations in the meta-analyses included in this review (see Fig 2). Part 2. Effect size calculations applied in our recalculations and subgroup meta-analyses (see Figs 3, 4 and S2). (PDF) [file pone.0169277.s002.pdf]

S1 Data. Overview of effect size calculations.

Part 1. Effect size calculations applied in the meta-analyses included in this review (see Fig 2).

When examining the difference between two conditions, effect size (ES) calculations based on standardized mean differences (SMD) are commonly recommended [1]. All meta-analyses included in this review reported SMD calculations, an overview of which is provided below.

1. SMD calculations reported by Schab et al. [2]:

"We employed the DerSimonian and Laird random effects model[58] with the standardized mean difference (SMD) as our measure of effect size (ES). The SMD describes the difference in outcome between the active and control arms of a trial in terms of the number of pooled standard deviations by which the two groups differ."

2. SMD calculations reported by Nigg et al. [3]:

"We computed effect sizes based on mean difference, standard deviation of differences, and across-condition correlation when those data were available."

"All effect sizes were converted to Hedges' g, a bias-free measure of standardized mean differences that can be interpreted in the same manner as the familiar Cohen's d."

"A random-effects model was chosen for analysis due to the likelihood that different studies would reflect different effect sizes based on their methods and samples."

3. SMD calculations reported by Benton [4]:

"Standardized mean differences (SMD) were calculated using the Hedges' g statistic under the fixed effects model. The heterogeneity statistic was then incorporated to calculate the summary standardized mean difference (SSMD) using the random effects model."

4: SMD calculations reported by Sonuga-Barke et al. [5]:

"Individual effect sizes (the standardized mean difference) were based on the recommended formula: mean pre- to post treatment change minus the mean pre- to post treatment control

group change divided by the pooled pretest standard deviation with a bias adjustment[23].”

“Given the heterogeneity of ADHD assessments, sample characteristics, and implementation of treatments within domains in the included studies, we chose a priori to use random-effects models, as recommended by Field and Gillett[27].”

5: SMD calculations reported by Gillies et al. [6]:

“We calculated the standardised mean difference (SMD) between groups and 95% CI using a fixed-effect model, or a random-effects model where there was significant heterogeneity. We used SMDs because more than one scale was used for each of these continuous outcomes.”

Please note that in Gillies et al.’s analyses 1.2 and 1.5 – which are the analyses included in this review – the following approach was reported: SMD, inverse variance, fixed model [6].

Part 2. Effect size calculations applied in our recalculations and subgroup meta-analyses (see Fig 3, Fig 4 and S2 Fig).

The Standardized Mean Difference (SMD) reported in Fig 3, Fig 4 and S2 Fig (i.e. Cohen’s d) was calculated using the metan command of STATA (version 14.1) and based on the following equation:

$$\mathrm{SMD}=\frac{M_c - M_t}{SD_{pooled}} \text{ where } SD_{pooled} = \sqrt{\frac{SD_c^2 * (n_c-1) + SD_t^2 * (n_t-1)}{(n_c+n_t-2)}}$$

M_c, M_t = mean of control and treatment group
SD_c, SD_t = standard deviation of control and treatment group
n_c, n_t = number of observations in control and treatment group

Cohen’s d is a practical and valuable effect size comparison measure [1, 7], often used in ADHD research to establish the clinical effect of an intervention [8-11]. We applied the random effects model – in which study weights are more balanced than in the fixed effect model – to estimate the mean effect size [12].

The % Weight reported in Fig 3, Fig 4 and in S1 and S2 Figs provides information on the contribution of each study to the meta-analytic outcomes, and has been calculated based on the inverse variance of each study's effect size.

References

1. Fritz CO, Morris PE, Richler JJ. Effect size estimates: current use, calculations, and interpretation. J Exp Psychol Gen. 2012;141(1):2-18.
2. Schab DW, Trinh NH. Do artificial food colors promote hyperactivity in children with hyperactive syndromes? A meta-analysis of double-blind placebo-controlled trials. J Dev Behav Pediatr. 2004;25(6):423-34.
3. Nigg JT, Lewis K, Edinger T, Falk M. Meta-analysis of attention-deficit/hyperactivity disorder or attention-deficit/hyperactivity disorder symptoms, restriction diet, and synthetic food color additives. J Am Acad Child Adolesc Psychiatry. 2012;51(1):86-97.e8.
4. Benton D. The impact of diet on anti-social, violent and criminal behaviour. Neurosci Biobehav Rev. 2007;31(5):752-74.
5. Sonuga-Barke EJ, Brandeis D, Cortese S, Daley D, Ferrin M, Holtmann M, et al. Nonpharmacological interventions for ADHD: systematic review and meta-analyses of randomized controlled trials of dietary and psychological treatments. Am J Psychiatry. 2013;170(3):275-89.
6. Gillies D, Sinn J, Lad SS, Leach MJ, Ross MJ. Polyunsaturated fatty acids (PUFA) for attention deficit hyperactivity disorder (ADHD) in children and adolescents. Cochrane Database Syst Rev. 2012;7:Cd007986.
7. Dunst CJ, Hamby DW. Guide for calculating and interpreting effect sizes and confidence intervals in intellectual and developmental disability research studies. J Intellect Dev Disabil. 2012;37(2):89-99.

8. Coghill D, Banaschewski T, Lecendreux M, Soutullo C, Johnson M, Zuddas A, et al. European, randomized, phase 3 study of lisdexamfetamine dimesylate in children and adolescents with attention-deficit/hyperactivity disorder. Eur Neuropsychopharmacol. 2013;23(10):1208-18.

9. Faraone SV, Buitelaar J. Comparing the efficacy of stimulants for ADHD in children and adolescents using meta-analysis. Eur Child Adolesc Psychiatry. 2010;19(4):353-64.

10. Hodgkins P, Shaw M, Coghill D, Hechtman L. Amfetamine and methylphenidate medications for attention-deficit/hyperactivity disorder: complementary treatment options. Eur Child Adolesc Psychiatry. 2012;21(9):477-92.

11.MTA Cooperative Group. A 14-month randomized clinical trial of treatment strategies for attention-deficit/hyperactivity disorder. Multimodal Treatment Study of Children with ADHD. Arch Gen Psychiatry. 1999;56(12):1073-86.

12. Borenstein M, Hedges LV, Higgins JP, Rothstein HR. Introduction to meta-analysis. John Wiley & Sons, Ltd, Chichester, 2009.
