# Supplementary material for: Diet and ADHD, Reviewing the Evidence: A Systematic Review of Meta-Analyses of Double-Blind Placebo-Controlled Trials Evaluating the Efficacy of Diet Interventions on the Behavior of Children with ADHD
Source: PLoS One. 2017 Jan 25;12(1):e0169277. doi: 10.1371/journal.pone.0169277 (PMC5266211; doi:10.1371/journal.pone.0169277)
Supplement: S1 Table — (PDF) [file pone.0169277.s003.pdf]

S1 Table. Characteristics of the most recently published reviews discussing the effect of diet on ADHD, and of the current review.

Characteristics	Stevenson [1] 2014	Nigg [2] 2014	Rytter [3] 2015	Current review
Search strategy	Not provided for AFC, FFD and PUFA studies; provided for studies on 'other supplements'	Not provided	Provided	Provided
Inclusion criteria	Provided for the 'other supplements' search only	Not provided	Provided	Provided
Language restrictions	Not provided	Not provided	English	No restrictions
Authors searching	Not provided	Not provided	Two researchers, independently	Two researchers, independently
Date search	End date provided for 'other supplements' search only	Not provided	Provided	Provided
Databases searched	Not provided	Not provided	Provided	Provided
Search terms	Not provided	Not provided	Provided	Provided
Further search strategies	Not provided	Not provided	Provided	Provided
All studies# solely included subjects meeting ADHD criteria	No	No	Yes	Yes
All studies# were DBPC trials	No	Yes	No	Yes
All studies# were controlled trials	Yes	Yes	No	Yes
All AFC studies# applied an AFC intervention	No	Yes	No	Yes
All FFD studies# applied a FFD intervention	No	Yes	Yes	Yes
All PUFA studies# applied a PUFA intervention	Yes	Yes	Yes	Yes
Referring to all AFC meta-analyses (n=3)^	Yes	Yes	Yes	Yes
Referring to all FFD meta-analyses (n=3)^	No	No	No	Yes
Referring to all PUFA meta-analyses (n=6)^	Yes (n=3)*	Yes (n=4)*	Yes (n=3)*	Yes (n=6)
Referring to the sugar meta-analysis (n=1)^	No	No	No	Yes
Referring to the Feingold meta-analysis (n=1)^	No	Yes	Yes	Yes
Referring to non-existing meta-analyses	Yes	Yes	No	No

AFC=artificial food colors; FFD=few-foods diet; PUFA=poly-unsaturated fatty acids; DBPC=double-blind placebo-controlled.
#Studies included in the meta-analyses evaluated [1,2] or in the systematic review [3].
^These meta-analyses resulted from the search strategy applied in the current review.
*3/6 or 2/6 not published yet at the time of publication of the review concerned.

References

1. Stevenson J, Buitelaar J, Cortese S, Ferrin M, Konofal E, Lecendreux M, et al. Research review: the role of diet in the treatment of attention-deficit/hyperactivity disorder--an appraisal of the evidence on efficacy and recommendations on the design of future studies. J Child Psychol Psychiatry. 2014;55(5):416-27.
2. Nigg JT, Holton K. Restriction and elimination diets in ADHD treatment. Child Adolesc Psychiatr Clin N Am. 2014;23(4):937-53.
3. Rytter MJ, Andersen LB, Houmann T, Bilenberg N, Hvolby A, Molgaard C, et al. Diet in the treatment of ADHD in children - a systematic review of the literature. Nord J Psychiatry. 2015;69(1):1-18.
