# Supplementary material for: Diet and ADHD, Reviewing the Evidence: A Systematic Review of Meta-Analyses of Double-Blind Placebo-Controlled Trials Evaluating the Efficacy of Diet Interventions on the Behavior of Children with ADHD
Source: PLoS One. 2017 Jan 25;12(1):e0169277. doi: 10.1371/journal.pone.0169277 (PMC5266211; doi:10.1371/journal.pone.0169277)
Supplement: S2 Table — (PDF) [file pone.0169277.s004.pdf]

S2 Table. Summary of characteristics of 14 meta-analyses evaluating diet interventions in ADHD, including reasons for exclusion (depicted by 'No').

First author Publication year (Number of studies)	Diet Intervention	Refers to previous meta-analyses	All studies are DBPC trials	All studies include children with ADHD/hyperactivity	All studies apply similar diet interventions	Included in this review
Wolraich [1] 1995 (n=23)	Eliminating one food group: Sugar	NA	Yes	No 18/23 studies non-ADHD subjects	Yes	No
Schab [2] 2004 (n=15)	Eliminating one food group: AFC	NA	Yes	Yes	Yes	Yes
Nigg [3] 2012 (n=11)	Eliminating one food group: AFC	Refers to Schab	Yes	Yes	Yes	Yes
Sonuga-Barke [4] 2013 (n=8)	Eliminating one food group: AFC	Refers to Nigg No referral to Schab	Yes*	Yes	No 2/8 studies are Feingold studies	No
Kavale [5] 1983 (n=23)	Eliminating some food groups: Feingold	NA	No 6/23 studies were not controlled	No 3/23 studies non-ADHD subjects	No 13/23 studies are AFC studies	No
Benton [6] 2007 (n=5)	Eliminating many food groups: FFD	NA	Yes	Yes	Yes	Yes
Nigg [3] 2012 (n=5)	Elimination many food groups: FFD	Not referring to Benton	Yes	Yes	No 2/5 studies are Feingold studies	No
Sonuga-Barke [4] 2013 (n=5)	Eliminating many food groups: FFD	Not referring to either Benton or Nigg	Yes*	Yes	Yes	Yes
Bloch [7] 2011 (n=10)	Supplementing PUFA	NA	Yes	No 2/10 studies non-ADHD subjects	Yes	No
Gillies [8] 2012 (n=9)	Supplementing PUFA	Refers to all previous PUFA meta-analyses	Yes	Yes	Yes	Yes
Sonuga-Barke [4] 2013 (n=11)	Supplementing PUFA	Refers to all previous PUFA meta-analyses	Yes*	Yes	Yes	Yes
Puri [9] 2014 (n=18)	Supplementing PUFA	Refers to all previous PUFA meta-analyses	Yes	No 4/18 studies non-ADHD subjects	Yes	No
Hawkey [10] 2014 (n=16)	Supplementing PUFA	Refers to all previous PUFA meta-analyses	Yes	No 5/16 studies non-ADHD subjects	Yes	No
Cooper [11] 2015 (n=24)	Supplementing PUFA	Refers to two previous PUFA meta-analyses [4,7]	No 1/24 studies not DBPC	No Studies in adults and in children without ADHD were included	Yes	No

AFC=artificial food color; FFD=few-foods diet; PUFA=poly-unsaturated fatty acid; DBPC=double-blind placebo-controlled.
*In this meta-analysis 'probably blinded' conditions were required instead of double-blind conditions. However, all studies included applied a DBPC design.

References

1. Wolraich ML, Wilson DB, White JW. The effect of sugar on behavior or cognition in children - a meta analysis. JAMA. 1995;274(20):1617-21.
2. Schab DW, Trinh NH. Do artificial food colors promote hyperactivity in children with hyperactive syndromes? A meta-analysis of double-blind placebo-controlled trials. J Dev Behav Pediatr. 2004;25(6):423-34.
3. Nigg JT, Lewis K, Edinger T, Falk M. Meta-analysis of attention-deficit/hyperactivity disorder or attention-deficit/hyperactivity disorder symptoms, restriction diet, and synthetic food color additives. J Am Acad Child Adolesc Psychiatry. 2012;51(1):86-97.e8.
4. Sonuga-Barke EJ, Brandeis D, Cortese S, Daley D, Ferrin M, Holtmann M, et al. Nonpharmacological interventions for ADHD: systematic review and meta-analyses of randomized controlled trials of dietary and psychological treatments. Am J Psychiatry. 2013;170(3):275-89.
5. Kavale KA, Forness SR. Hyperactivity and diet treatment: a meta-analysis of the Feingold hypothesis. J Learn Disabil. 1983;16(6):324-30.
6. Benton D. The impact of diet on anti-social, violent and criminal behaviour. Neurosci Biobehav Rev. 2007;31(5):752-74.
7. Bloch MH, Qawasmi A. Omega-3 fatty acid supplementation for the treatment of children with attention-deficit/hyperactivity disorder symptomatology: systematic review and meta-analysis. J Am Acad Child Adolesc Psychiatry. 2011;50(10):991-1000.
8. Gillies D, Sinn J, Lad SS, Leach MJ, Ross MJ. Polyunsaturated fatty acids (PUFA) for attention deficit hyperactivity disorder (ADHD) in children and adolescents. Cochrane Database Syst Rev. 2012;7:Cd007986. Epub 2012/07/13.
9. Puri BK, Martins JG. Which polyunsaturated fatty acids are active in children with attention-deficit hyperactivity disorder receiving PUFA supplementation? A fatty acid validated meta-regression analysis of randomized controlled trials. Prostaglandins Leukot Essent Fatty Acids. 2014;90(5):179-89.
10. Hawkey E, Nigg JT. Omega-3 fatty acid and ADHD: blood level analysis and meta-analytic extension of supplementation trials. Clin Psychol Rev. 2014;34(6):496-505.
11. Cooper RE, Tye C, Kuntsi J, Vassos E, Asherson P. Omega-3 polyunsaturated fatty acid supplementation and cognition: A systematic review and meta-analysis. Journal of psychopharmacology (Oxford, England). 2015;29(7):753-63.
