# Supplementary material for: Diet and ADHD, Reviewing the Evidence: A Systematic Review of Meta-Analyses of Double-Blind Placebo-Controlled Trials Evaluating the Efficacy of Diet Interventions on the Behavior of Children with ADHD
Source: PLoS One. 2017 Jan 25;12(1):e0169277. doi: 10.1371/journal.pone.0169277 (PMC5266211; doi:10.1371/journal.pone.0169277)
Supplement: S3 Table — (PDF) [file pone.0169277.s005.pdf]

S3 Table. Overview of FFD RCTs' raters, design, and of the data provided in the articles (either graphical or in figures) and included in Fig 3, Fig 4 and S2 Fig (denoted by '1').

			Data				Figure				
			T		C						
Author	Rater	Design, intervention	n	mean (sd)	n	mean (sd)	3A	3B	4A	4B	S2
Kaplan [1] 1989	Parent	DBPC Diet	24	10.8 (4.2)	24	13.1 (4.1)	1	1			1
Kaplan [1] 1989	Daycare	DBPC Diet	10	10.73 (6.51)	10	13.07 (6.97)			1	1	1
Schmidt [2] 1997	Ward, play observation	DBPC Diet	49	18.5 (9.0)	49	22.8 (9.7)	1	1			1
Schmidt [2] 1997	Test observation	DBPC Diet	49	14 (8.9)	49	17.2 (9.2)			1	1	1
Egger [3] 1985	Parent	DBPC Challenge	25	6.6 (4.4)	25	11.9 (15.7)	1	1	1	1	1
Carter [4] 1993	Parent	DBPC Challenge	19	8.8 (7.4)	19	13.9 (9.1)	1	1			1
Carter [4] 1993	Test observation	DBPC Challenge	19	0.57 (0.4)	19	0.8 (0.4)			1	1	1
Boris [5] 1994	Parent	DBPC Challenge	16	8.2 (5.32)	16	18.1 (6.96)	1	1	1	1	1

FFD=few-foods diet; RCT=randomized controlled trial; DBPC=double-blind placebo-controlled; T=treatment (FFD or placebo challenge); C=control (placebo diet, active challenge or waitlist); n=number of children included; mean=mean score at end intervention; sd=standard deviation of the mean.

References

1. Kaplan BJ, McNicol J, Conte RA, Moghadam HK. Dietary replacement in preschool-aged hyperactive boys. Pediatrics. 1989;83(1):7-17.
2. Schmidt MH, Mocks P, Lay B, Eisert HG, Fojkar R, Fritz-Sigmund D, et al. Does oligoantigenic diet influence hyperactive/conduct-disordered children--a controlled trial. Eur Child Adolesc Psychiatry. 1997;6(2):88-95.
3. Egger J, Carter CM, Graham PJ, Gumley D, Soothill JF. Controlled trial of oligoantigenic treatment in the hyperkinetic syndrome. Lancet. 1985;1(8428):540-5.
4. Carter CM, Urbanowicz M, Hemsley R, Mantilla L, Strobel S, Graham PJ, et al. Effects of a few food diet in attention deficit disorder. Arch Dis Child. 1993;69(5):564-8. Epub 1993/11/01. PubMed PMID: 8257176; PubMed Central PMCID: PMCPmc1029619.
5. Boris M, Mandel FS. Foods and additives are common causes of the attention deficit hyperactive disorder in children. Ann Allergy. 1994;72(5):462-8.
