# Supplementary material for: Diet and ADHD, Reviewing the Evidence: A Systematic Review of Meta-Analyses of Double-Blind Placebo-Controlled Trials Evaluating the Efficacy of Diet Interventions on the Behavior of Children with ADHD
Source: PLoS One. 2017 Jan 25;12(1):e0169277. doi: 10.1371/journal.pone.0169277 (PMC5266211; doi:10.1371/journal.pone.0169277)
Supplement: S1 Fig — (see Sonuga-Barke et al.’s page 283, Fig 3A), except for the teacher data provided by Schmidt et al., which are replaced by the test observation data. Forest plot of FFD effects and homogeneity statistics. (PDF) [file pone.0169277.s006.pdf]

S1 Figure

S1 Fig. Data provided in the probably blinded assessments' few-foods diet (FFD) meta-analysis by Sonuga-Barke et al. [1] (see Sonuga-Barke et al.'s page 283 Figure 3.A), except for the teacher data provided by Schmidt et al. [2], which are replaced by the test observation data. Forest plot of FFD effects and homogeneity statistics.

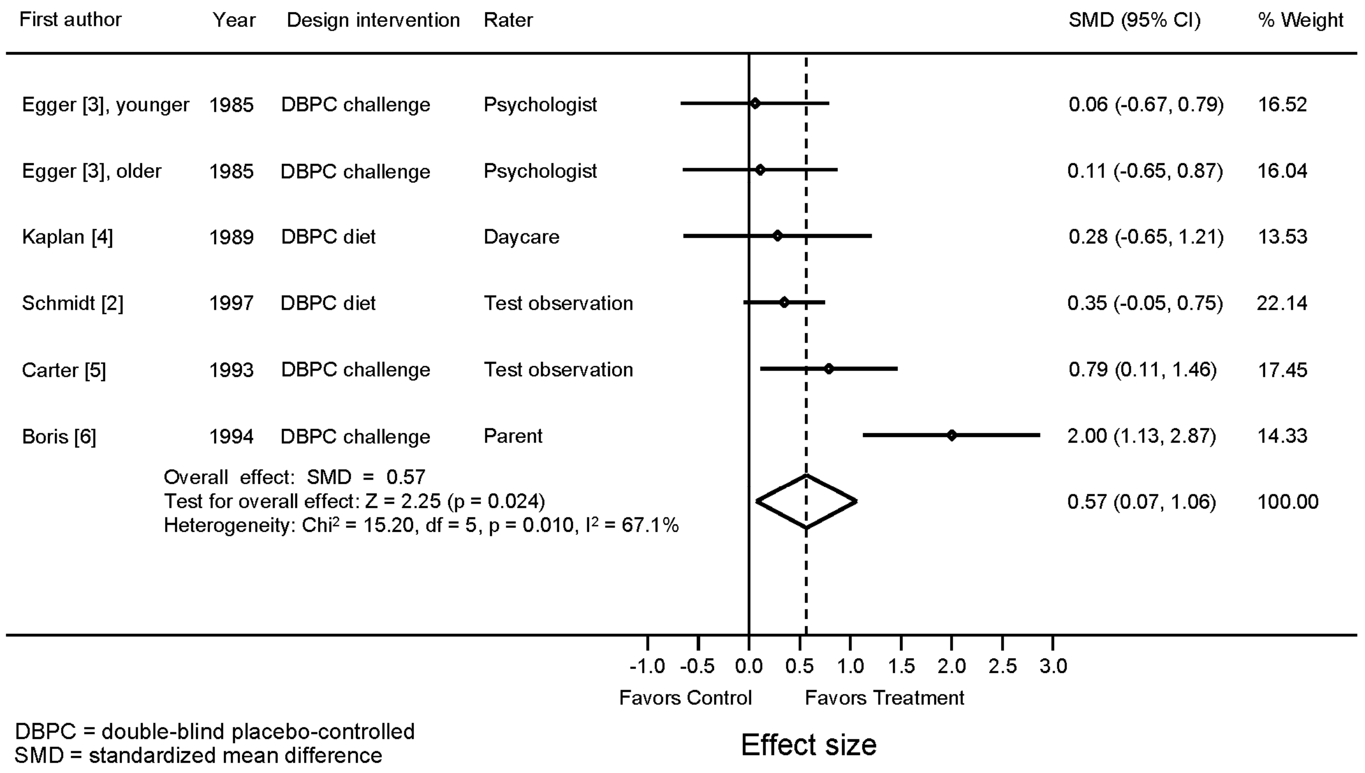

In S1 Fig the rating results as reported by Sonuga-Barke et al. [1] were included, except for Schmidt et al.'s teacher ratings results, which were replaced by the test observation rating results [2]. Since Sonuga-Barke et al. supplied graphical results only (see Figure 3.A, page 283 of Sonuga-Barke et al.'s paper [1]) we used MS Publisher's ruler to obtain the effect size and lower and upper level of the 95% CI of the blinded measurement data. For three reasons we replaced the teacher measurements provided in the fifth study [2] and included by Sonuga-Barke et al. [1], by the test observation measurements also provided by Schmidt et al. [2]:

First, the teacher ratings provided by Schmidt et al. [2] appeared to be biased. These ratings were generated in a specialist setting and could not even detect behavioral problems at baseline (see page 92, Table 2, of Schmidt et al.'s paper [2]). Probably, as has been suggested by Sonuga-Barke et al. [7], specialized teacher settings might be therapeutically beneficial already. In addition, 'classroom ratings were not even able to demonstrate the well-established treatment effects of stimulant therapy' [2]. For good reason Schmidt et al. excluded these ratings from further analyses [2].

Second, according to the guidelines described by Sonuga-Barke et al. [1] observer ratings should be considered 'better probably blinded' than teacher ratings, and thus, if both ratings were available, the observer ratings should be included (see also Results, section 'Sub-analysis based on the FFD meta-analysis by Sonuga-Barke et al.', bullet point 2).

Third, reproducing the meta-analytic calculations of Sonuga-Barke et al. [1] including Schmidt et al.'s test observation measurements rather than the specialized teacher ratings [2] may reduce bias in the meta-analytic estimates.

The calculations provided in S1 Fig resulted in an effect size of 0.57 (95% CI: 0.07–1.06, $p = 0.024$, $I^2 = 67\%$), which is commensurate with the results of Sonuga-Barke et al. [1] (ES = 0.51, 95% CI: -0.02–1.04, $p = 0.06$, $I_2 = 72\%$). However, if an intervention's efficacy is inferred from p-values being < 0.05,

then the inclusion of either Schmidt et al.'s teacher ratings (p = 0.06) or test observation ratings (p = 0.024) would potentially generate different conclusions. The following three statements in the abstract of Sonuga-Barke et al.'s paper [1] suggest that statistical effects are considered more important than clinical effects:

1) The significant effects of most treatments attenuated to non-significant levels when evaluating the blinded ratings only,
2) PUFA supplementation (ES = 0.16, p = 0.04) 'produced small but significant reductions in ADHD symptoms even with probably blinded assessments, although the clinical significance of these effects remains to be determined',
3) 'Better evidence for efficacy from blinded assessments is required' for a FFD (ES = 0.51, p = 0.06) before this intervention 'can be supported as treatments for core ADHD symptoms'.

These statements convey that, although effect sizes are reported as well, the conclusions are p-value based. Comparison of the results reported in S1 Fig and in Figure 3.A. provided by Sonuga-Barke et al. [1], illustrates the limitation of relying on p-values, a topic which is further addressed in the discussion section (see 'Differences in conclusions between our and previous reviews').

References

1. Sonuga-Barke EJ, Brandeis D, Cortese S, Daley D, Ferrin M, Holtmann M, et al. Nonpharmacological interventions for ADHD: systematic review and meta-analyses of randomized controlled trials of dietary and psychological treatments. Am J Psychiatry. 2013;170(3):275-89.
2. Schmidt MH, Mocks P, Lay B, Eisert HG, Fojkar R, Fritz-Sigmund D, et al. Does oligoantigenic diet influence hyperactive/conduct-disordered children--a controlled trial. Eur Child Adolesc Psychiatry. 1997;6(2):88-95.
3. Egger J, Carter CM, Graham PJ, Gumley D, Soothill JF. Controlled trial of oligoantigenic treatment in the hyperkinetic syndrome. Lancet. 1985;1(8428):540-5.
4. Kaplan BJ, McNicol J, Conte RA, Moghadam HK. Dietary replacement in preschool-aged hyperactive boys. Pediatrics. 1989;83(1):7-17.
5. Carter CM, Urbanowicz M, Hemsley R, Mantilla L, Strobel S, Graham PJ, et al. Effects of a few food diet in attention deficit disorder. Arch Dis Child. 1993;69(5):564-8.
6. Boris M, Mandel FS. Foods and additives are common causes of the attention deficit hyperactive disorder in children. Ann Allergy. 1994;72(5):462-8.
7. Sonuga-Barke EJ, Van Lier P, Swanson JM, Coghill D, Wigal S, Vandenberghe M, et al. Heterogeneity in the pharmacodynamics of two long-acting methylphenidate formulations for children with attention deficit/hyperactivity disorder. Ann Allergy. 1994;72(5):462-8.
