# Supplementary material for: Diet and ADHD, Reviewing the Evidence: A Systematic Review of Meta-Analyses of Double-Blind Placebo-Controlled Trials Evaluating the Efficacy of Diet Interventions on the Behavior of Children with ADHD
Source: PLoS One. 2017 Jan 25;12(1):e0169277. doi: 10.1371/journal.pone.0169277 (PMC5266211; doi:10.1371/journal.pone.0169277)
Supplement: S2 Fig — Forest plot of FFD effects and homogeneity statistics. (PDF) [file pone.0169277.s007.pdf]

S2 Figure

S2 Fig. Sub-analysis of DBPC RCTs applying a few-foods diet (FFD) intervention, subgrouped per rater (parent or other). Forest plot of FFD effects and homogeneity statistics.

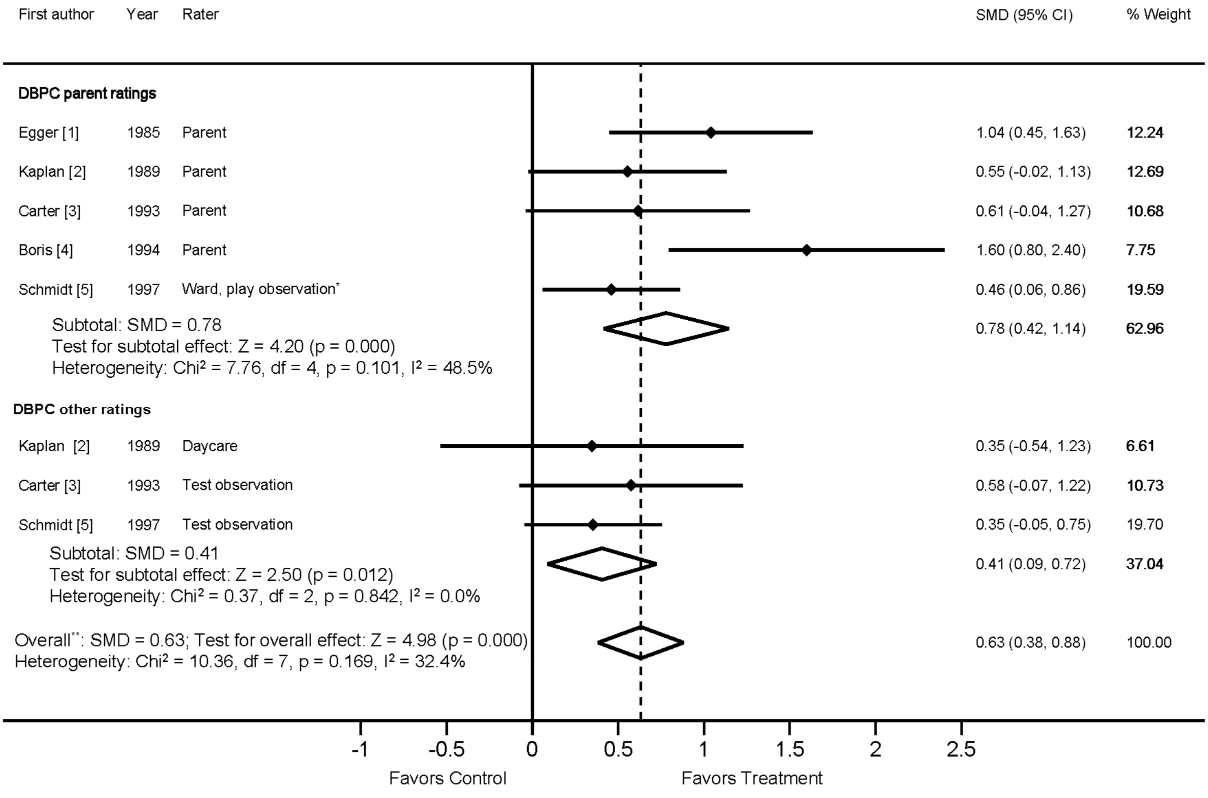

DBPC = double-blind placebo-controlled
SMD = standardized mean difference

* inpatient study: ward ratings are commensurable to parent ratings
** after adjusting for multiple observations per study: SMD = 0.59, 95% CI: (0.06, 1.13), p = 0.029

References

1. Egger J, Carter CM, Graham PJ, Gumley D, Soothill JF. Controlled trial of oligoantigenic treatment in the hyperkinetic syndrome. Lancet. 1985;1(8428):540-5.
2. Kaplan BJ, McNicol J, Conte RA, Moghadam HK. Dietary replacement in preschool-aged hyperactive boys. Pediatrics. 1989;83(1):7-17.
3. Carter CM, Urbanowicz M, Hemsley R, Mantilla L, Strobel S, Graham PJ, et al. Effects of a few food diet in attention deficit disorder. Arch Dis Child. 1993;69(5):564-8. Epub 1993/11/01. PubMed PMID: 8257176; PubMed Central PMCID: PMCPmc1029619.
4. Boris M, Mandel FS. Foods and additives are common causes of the attention deficit hyperactive disorder in children. Ann Allergy. 1994;72(5):462-8.
5. Schmidt MH, Mocks P, Lay B, Eisert HG, Fojkar R, Fritz-Sigmund D, et al. Does oligoantigenic diet influence hyperactive/conduct-disordered children--a controlled trial. Eur Child Adolesc Psychiatry. 1997;6(2):88-95.
