# Supplementary material for: Diet and ADHD, Reviewing the Evidence: A Systematic Review of Meta-Analyses of Double-Blind Placebo-Controlled Trials Evaluating the Efficacy of Diet Interventions on the Behavior of Children with ADHD
Source: PLoS One. 2017 Jan 25;12(1):e0169277. doi: 10.1371/journal.pone.0169277 (PMC5266211; doi:10.1371/journal.pone.0169277)
Supplement: S1 Text — (PDF) [file pone.0169277.s008.pdf]

S1 Text. Strengths and weaknesses of double-blind placebo-controlled RCTs investigating the effect of a few-foods diet on ADHD.

Five randomized controlled trials (RCTs), investigating the impact of a few-foods diet (FFD) on ADHD, applied a double-blind placebo-controlled (DBPC) design [1-5]. Given that an optimal FFD consists of an average of six different foods each day, e.g. rice, turkey, vegetable, fruit, fat [3], while diet-as-usual comprises at least 30 different daily foods [6], reliable blinding (i.e. disguising both verum and placebo diet to such an extent that none of the persons involved are able to distinguish which of both diets is supposed to be the verum diet) is hardly conceivable. Consequently, in all DBPC FFD studies the diet was adapted in order to secure the blinding.

Two of five studies, performed in unselected groups of children – one of which in an inpatient group [5] – applied a DBPC *diet* design, i.e. comparing a FFD to a placebo diet [2, 5]. In these studies the researchers had to elaborate the verum diet and to restrict the placebo diet in order to conceal treatment conditions. Hence, the FFD comprised more than six foods and the restricted placebo diet was not equivalent to the child's diet-as-usual. Comparison of a more restricted FFD to diet-as-usual might have generated different results, but would also have jeopardized the blinding [2].

Three of five studies applied a DBPC *challenge* design [1, 3, 4] in responders selected previous to randomization. First, unselected groups of children followed an open FFD, after which the diet responders proceeded with an open challenge phase during which one food a week was reintroduced in normal amounts [3] to identify the incriminated foods. Subsequently, based on the results of the open challenges, a placebo was constructed for each food that was associated with behavioral deterioration and that could be disguised in foods not associated with behavioral problems. Finally, a DBPC food challenge was done in order to verify the open findings. It is self-evident that a DBPC food challenge, applying an

intervention that might establish the effect of single foods on behavior, would not be feasible without responder selection preceding the challenge: haphazardly challenging low doses of a random food would be unavailing and unethical since each child may respond to different foods [1, 3, 4].

To assure the blinding, the quantities of the challenged foods had to be restricted [1, 3, 4], because the amounts of foods that can be concealed are limited – e.g. to disguise 78 ml of cow milk, 250 ml test food is required [7]. Furthermore, each DBPC challenge included one food only, while the open challenge phase had shown that almost all responders reacted to more than one food [1, 3, 4]. Finally, the suboptimal intervention conditions in the challenge studies, i.e. the limited doses and numbers of food challenged, may have affected the study results [1, 3, 4].

References

1. Egger J, Carter CM, Graham PJ, Gumley D, Soothill JF. Controlled trial of oligoantigenic treatment in the hyperkinetic syndrome. Lancet. 1985;1(8428):540-545.
2. Kaplan BJ, McNicol J, Conte RA, Moghadam HK. Dietary replacement in preschool-aged hyperactive boys. Pediatrics. 1989;83(1):7-17.
3. Carter CM, Urbanowicz M, Hemsley R, Mantilla L, Strobel S, Graham PJ, et al. Effects of a few food diet in attention deficit disorder. Arch Dis Child. 1993;69(5):564-568.
4. Boris M, Mandel FS. Foods and additives are common causes of the attention deficit hyperactive disorder in children. Ann Allergy. 1994;72(5):462-468.
5. Schmidt MH, Mocks P, Lay B, Eisert HG, Fojkar R, Fritz-Sigmund D, et al. Does oligoantigenic diet influence hyperactive/conduct-disordered children--a controlled trial. Eur Child Adolesc Psychiatry. 1997;6(2):88-95.
6. Benton D. The influence of children's diet on their cognition and behavior. Eur J Nutr. 2008;47 Suppl 3:25-37.
7. Vlieg-Boerstra BJ, Herpertz I, Pasker L, van der Heide S, Kukler J, Jansink C, et al. Validation of novel recipes for double-blind, placebo-controlled food challenges in children and adults. Allergy. 2011;66(7):948-954.
