# Supplementary material for: Diet and ADHD, Reviewing the Evidence: A Systematic Review of Meta-Analyses of Double-Blind Placebo-Controlled Trials Evaluating the Efficacy of Diet Interventions on the Behavior of Children with ADHD
Source: PLoS One. 2017 Jan 25;12(1):e0169277. doi: 10.1371/journal.pone.0169277 (PMC5266211; doi:10.1371/journal.pone.0169277)
Supplement: S2 Text — (PDF) [file pone.0169277.s009.pdf]

S2 Text. Proposition for an unambiguous nomenclature of diet research.

Current nomenclature of diet research in ADHD is divergent and confusing. A practical classification has been provided in an overview of 35 years diet research in ADHD [1] differentiating between three types of diet intervention studies, all of them *eliminating* foods from the actual diet: 1) artificial food color (AFC) studies eliminating one group of foods, i.e. additives; 2) Feingold studies eliminating some groups of foods, i.e. colors, flavors and foods rich in salicylates, and 3) few-foods diet (FFD) studies eliminating many foods and additives, i.e. allowing a few foods only. Supplement studies, i.e. investigating the effect of adding nutrients to the diet, were not included in this overview [1]. Considering the apparent differences between diet supplement research (adding some nutrients to the habitual diet without changing the actual food intake) and diet elimination research (eliminating foods from the habitual diet, consequently changing the daily food intake) we propose to divide diet research in ADHD into two groups: *supplement research* and *elimination research*.

Supplement research is generally named after the supplement that is investigated. Conversely, elimination research nomenclature is characterized by heterogeneity: The tripartite classification by Stevens et al. [1] based on the number of foods eliminated (i.e. AFC studies; Feingold studies; FFD studies), was adopted by some authors [2], while others divided the intervention studies in slightly different categories (i.e. AFC studies; sugar studies; FFD studies) [3]. A third group of researchers used the designation restriction/elimination diet to cover any type of dietary restriction regardless of the number of foods eliminated [4-6], while a fourth group applied the same designation (i.e. restricted elimination) to refer to the FFD [7, 8]. In order to provide researchers and patients with the utmost transparency about the kind of elimination diet applied, we propose to classify elimination studies as follows:

1) When excluding one food, food group or component only, the study should be named commensurately (i.e. 'additives study' when investigating the effect of additives only or 'sugar study' when investigating the effect of sugar only);

2) When excluding some foods or food groups (like the Feingold diet or like a diet eliminating allergenic and high-histamine food groups) the study should be called 'elimination study', specifying the *eliminated* foods and food groups in the abstract;

3) When excluding a great many foods, food groups and additives, the denomination 'FFD study' is appropriate, specifying the *permitted* foods in the abstract.

References

1. Stevens LJ, Kuczek T, Burgess JR, Hurt E, Arnold LE. Dietary sensitivities and ADHD symptoms: thirty-five years of research. Clin Pediatr (Phila). 2011;50(4):279-293.
2. Verlaet AA, Noriega DB, Hermans N, Savelkoul HF. Nutrition, immunological mechanisms and dietary immunomodulation in ADHD. Eur Child Adolesc Psychiatry. 2014;23(7):519-529.
3. Rytter MJ, Andersen LB, Houmann T, Bilenberg N, Hvolby A, Molgaard C, et al. Diet in the treatment of ADHD in children - a systematic review of the literature. Nord J Psychiatry. 2015;69(1):1-18.
4. Arnold LE, Hurt E, Lofthouse N. Attention-deficit/hyperactivity disorder: dietary and nutritional treatments. Child Adolesc Psychiatr Clin N Am. 2013;22(3):381-402, v.
5. Millichap JG, Yee MM. The diet factor in attention-deficit/hyperactivity disorder. Pediatrics. 2012;129(2):330-337.
6. Nigg JT, Holton K. Restriction and elimination diets in ADHD treatment. Child Adolesc Psychiatr Clin N Am. 2014;23(4):937-953.
7. Sonuga-Barke EJ, Brandeis D, Cortese S, Daley D, Ferrin M, Holtmann M, et al. Nonpharmacological interventions for ADHD: systematic review and meta-analyses of randomized controlled trials of dietary and psychological treatments. Am J Psychiatry. 2013;170(3):275-289.
8. Stevenson J, Buitelaar J, Cortese S, Ferrin M, Konofal E, Lecendreux M, et al. Research review: the role of diet in the treatment of attention-deficit/hyperactivity disorder--an appraisal of the evidence on efficacy and recommendations on the design of future studies. J Child Psychol Psychiatry. 2014;55(5):416-427.
