# Supplementary material for: Diet and ADHD, Reviewing the Evidence: A Systematic Review of Meta-Analyses of Double-Blind Placebo-Controlled Trials Evaluating the Efficacy of Diet Interventions on the Behavior of Children with ADHD
Source: PLoS One. 2017 Jan 25;12(1):e0169277. doi: 10.1371/journal.pone.0169277 (PMC5266211; doi:10.1371/journal.pone.0169277)
Supplement: S3 Text — (PDF) [file pone.0169277.s010.pdf]

S3 Text. Proposition for unambiguous definitions of blinding.

Sonuga-Barke et al. [1] introduced three unusual blinding definitions, i.e. *probably blinded, better probably blinded* and *best probably blinded.* In Sonuga-Barke et al.'s meta-analyses depicted in Figure 3 [1] (evaluating the overall effect of Restricted Elimination Diets, Artificial Food Color Exclusions, Supplementation With Free Fatty Acids, Cognitive Training, Neurofeedback and Behavioral Interventions), the designation *probably blinded* was used for assessments made in 'both placebo- and non-placebo-controlled trials', i.e. 'either ratings clearly made under blind conditions' (e.g. resulting from placebo-controlled trials), 'or ratings made by an adult unlikely to be aware of treatment allocation' (e.g. resulting from non-placebo or non-blinded, open randomized controlled trials (RCTs) [1]. In open trials the ADHD assessments made by parents in school-implemented interventions and by teachers in home-implemented interventions were considered *probably blinded*, while observer ratings were considered the *best probably blinded* assessments [1]. In double-blind placebo-controlled (DBPC) trials all ratings were considered *probably blinded*, while observer and teacher ratings (in that order of preference) were considered *better probably blinded* in DBPC home-implemented interventions [1]. In trials in which more than one *probably blinded* measure was available, the best *probably blinded* measure was selected to be included in the meta-analyses of probably blinded assessments [1]. In our opinion this aberrant blinding nomenclature is confusing:

First, in open RCTs with a JADAD score for blinding of zero – whether the intervention is home-implemented or school implemented – it is self-evident that all ratings are *not blinded* (please note that the maximum JADAD score for blinding is two, indicating that the blinding method was both described and appropriate, and that neither participants nor assessors (whether parents, teachers or observers) were able to identify the intervention applied [2]). However, in Sonuga-Barke et al.'s Figure 3F, depicting the probably blinded

assessments' meta-analysis of Behavioral Interventions [1], five of seven RCTs included received a JADAD score for blinding of zero (see section 2 of Sonuga-Barke et al.'s data supplement [1]), indicating that all raters were aware of or able to identify the intervention applied. Indeed, it would be quite probable that observers providing open home observation ratings in an un-blinded home-implemented parent training intervention [3] and teachers providing open teacher ratings in an un-blinded parent and child training intervention [4] are aware of the intervention applied, because parents are highly likely to impart their knowledge with teachers or observers, especially when not specifically instructed not to do so. In sum, denoting un-blinded ratings as *probably blinded* suggests a probability of blinding that in reality does not exist.

Second, in DBPC RCTs all ratings are 'clearly made under blind conditions' [1]. In studies receiving the highest JADAD score all raters are equally blinded – none of them being able to identify the intervention given [2] – which applies to 5/8 artificial food color studies, 5/5 few-foods diet studies and 8/11 fatty acid studies included in Sonuga-Barke et al.'s probably blinded assessments' meta-analyses (see Figures 3B, 3A, 3C and section 2 of the data supplement provided by Sonuga-Barke et al. [1]). Denoting clearly blinded ratings as *probably blinded* would not do justice to these studies, being properly blinded. Furthermore, labeling observer and teacher measurements (in that order of preference) provided in DBPC trials as *better probably blinded* wrongfully suggests that parents would be less blinded compared to other raters. Actually, the designation 'better (probably) blinded' is a contradiction in terms: if 'better' (probably) blinded is possible, the blinding is not accurate.

Third, the *probably blinded and better probably blinded assessments'* definitions are difficult to apply, even by the authors introducing these definitions [1]. For example, contrary to the above-described direction to prefer observer ratings to teacher ratings in home-implemented interventions with a DBPC design, Sonuga-Barke et al. included teacher ratings in the Restricted Elimination Diets meta-analysis (Figure 3A) [1], despite the availability of

observer ratings [5]. Likewise, in their Cognitive Training meta-analysis (Figure 3D) [1] teacher ratings resulting from a school-implemented open trial [6] were included instead of parent ratings. Finally, considering that open teacher ratings resulting from home-implemented un-blinded behavioral RCTs were eligible to be included in the probably blinded assessments' meta-analysis of Behavioral Interventions (Figure 3F) [1], the equally open teacher ratings resulting from home-implemented un-blinded diet RCTs [7, 8] might have been included in the probably blinded assessments' meta-analysis of Restricted Elimination Diets (Figure 3A) [1] as well.

In concert, the three newly introduced blinding definitions (*probably blinded, better probably blinded and best probably blinded assessments)* are confusing, are likely to engender fallacies and may be easily misconceived. In our view blinding should not be a matter of distance or place, but of method and design. To provide researchers and readers with the utmost clarity when blinding is concerned, we suggest that:

1) The designation 'blinded' should be used for all ratings in studies with a double-blind design and a JADAD score of two;

2) The designation 'probably blinded' should be used for ratings in studies with a JADAD score of one;

3) The designation 'not blinded' should be used for all ratings in studies with a JADAD score of zero, i.e. open trials.

References

1. Sonuga-Barke EJ, Brandeis D, Cortese S, Daley D, Ferrin M, Holtmann M, et al. Nonpharmacological interventions for ADHD: systematic review and meta-analyses of randomized controlled trials of dietary and psychological treatments. Am J Psychiatry. 2013;170(3):275-289.
2. Jadad AR, Moore RA, Carroll D, Jenkinson C, Reynolds DJ, Gavaghan DJ, et al. Assessing the quality of reports of randomized clinical trials: is blinding necessary? Control Clin Trials. 1996;17(1):1-12.
3. Pisterman S, Firestone P, McGrath P, Goodman JT, Webster I, Mallory R, et al. The role of parent training in treatment of preschoolers with ADDH. Am J Orthopsychiatry. 1992;62(3):397-408.
4. Webster-Stratton CH, Reid MJ, Beauchaine T. Combining parent and child training for young children with ADHD. J Clin Child Adolesc Psychol. 2011;40(2):191-203.
5. Schmidt MH, Mocks P, Lay B, Eisert HG, Fojkar R, Fritz-Sigmund D, et al. Does oligoantigenic diet influence hyperactive/conduct-disordered children--a controlled trial. Eur Child Adolesc Psychiatry. 1997;6(2):88-95.
6. Steiner NJ, Sheldrick RC, Gotthelf D, Perrin EC. Computer-based attention training in the schools for children with attention deficit/hyperactivity disorder: a preliminary trial. Clin Pediatr (Phila). 2011;50(7):615-622.
7. Pelsser LM, Frankena K, Toorman J, Savelkoul HF, Dubois AE, Pereira RR, et al. Effects of a restricted elimination diet on the behaviour of children with attention-deficit hyperactivity disorder (INCA study): a randomised controlled trial. Lancet. 2011;377(9764):494-503.
8. Pelsser LM, Frankena K, Toorman J, Savelkoul HF, Pereira RR, Buitelaar JK. A randomised controlled trial into the effects of food on ADHD. Eur Child Adolesc Psychiatry. 2009;18(1):12-19.
